# An Intervention Program to Reduce Medication-Related Problems Among Polymedicated Home-Dwelling Older Adults (OptiMed): Protocol for a Pre-Post, Multisite, Pilot, and Feasibility Study

**DOI:** 10.2196/39130

**Published:** 2023-01-25

**Authors:** Filipa Pereira, Maria dos Anjos Dixe, Sónia Gonçalves Pereira, Carla Meyer-Massetti, Henk Verloo

**Affiliations:** 1 School of Health Sciences University of Applied Sciences (HES-SO) Valais/Wallis Sion Switzerland; 2 Abel Salazar Institute of Biomedical Sciences University of Porto Porto Portugal; 3 Center for Innovative Care and Health Technology Polytechnic of Leiria Leiria Portugal; 4 Institute for Primary Health Care BIHAM University of Bern Bern Switzerland; 5 Clinical Pharmacology and Toxicology Department of General Internal Medicine Inselspital, University Hospital of Bern Bern Switzerland; 6 Service of Old Age Psychiatry Lausanne University Hospital Lausanne Switzerland

**Keywords:** protocol, pilot study, feasibility, medication safety, medication-related problems, medication management, home-dwelling older adults, informal caregivers, interprofessional collaboration, patient-centered care, quasi-experimental, pre-post study, polypharmacy

## Abstract

**Background:**

Effective medication management is one of the essential preconditions for enabling polymedicated home-dwelling older adults with multiple chronic conditions to remain at home and preserve their quality of life and autonomy. Lack of effective medication management predisposes older adults to medication-related problems (MRPs) and adverse health outcomes, which can lead to the degradation of a patient’s acute clinical condition, physical and cognitive decline, exacerbation of chronic medical conditions, and avoidable health care costs. Nonetheless, it has been shown that MRPs can be prevented or reduced by using well-coordinated, patient-centered, interprofessional primary care interventions.

**Objective:**

This study aimed to explore the feasibility and acceptability of an evidence-based, multicomponent, interprofessional intervention program supported by informal caregivers to decrease MRPs among polymedicated home-dwelling older adults with multiple chronic conditions.

**Methods:**

This quasi-experimental, pre-post, multisite pilot, and feasibility study will use an open-label design, with participants knowing the study’s objectives and relevant information, and it will take place in primary health care settings in Portugal and Switzerland. The research population will comprise 30 polymedicated, home-dwelling adults, aged ≥65 years at risk of MRPs and receiving community-based health care, along with their informal caregivers and health care professionals.

**Results:**

Before a projected full-scale study, this pilot and feasibility study will focus on recruiting and ensuring the active collaboration of its participants and on the feasibility of expanding this evidence-based, multicomponent, interprofessional intervention program throughout both study regions. This study will also be essential to projected follow-up research programs on informal caregivers’ multiple roles, enhancing their coordination tasks and their own needs. Results are expected at the end of 2024.

**Conclusions:**

Designing, establishing, and exploring the feasibility and acceptability of an intervention program to reduce the risks of MRPs among home-dwelling older adults is an underinvestigated issue. Doing so in collaboration with all the different actors involved in that population’s medication management and recording the first effects of the intervention will make this pilot and feasibility study’s findings very valuable as home care becomes an ever more common solution.

**Trial Registration:**

Swiss National Clinical Trials Portal 000004654; https://tinyurl.com/mr3yz8t4

## Introduction

### Background

Older adults with multiple chronic health conditions—particularly those with cognitive impairment—frequently depend on complex medication regimens [1]. Although the use of ≥5 medications, known as polypharmacy [2], may be clinically appropriate in many cases, inappropriate prescriptions may put older adults at a greater risk of medication-related problems (MRPs) and adverse health outcomes [3,4]. MRPs can lead to degradation in a patient’s acute clinical condition, physical and cognitive decline, an exacerbation of chronic medical conditions, and avoidable health care costs [5,6]. This public health issue has been highlighted in various European countries, including Portugal and Switzerland [7,8].

Nonetheless, MRPs have been demonstrated to be preventable or reducible using well-coordinated, patient-centered, interprofessional primary care [4,5,9]. Current evidence-based strategies for medication safety and the prevention of MRPs include medication reconciliation, systematic and comprehensive reviews of prescribed medications (including deprescribing), and electronic medication management systems [10,11]. Medication reconciliation is a well-documented evidence-based practice involving creating and updating a single list of an older adult’s currently prescribed medications [12] and then systematically and comprehensively reviewing them. Prior research has shown that hospital discharge documents received by primary care professionals are frequently inaccurate and lack key information [13]. Regular medication reconciliation reduces medication errors owing to consistent communication during transitions of care, including changes in clinical settings, medical practitioners, or the level of care [14]. Regularly and systematically reviewing prescribed medications is a straightforward, effective means of reducing inappropriate prescribing and prescribing cascades and is, therefore, a preventive strategy for avoiding MRPs [15]. Inappropriate prescribing includes potentially inappropriate medications and potential prescribing omissions [15]. Existing assessment tools—such as the STOPP (Screening Tool of Older Persons’ Prescriptions) and START (Screening Tool to Alert to Right Treatment) criteria—recognize the dual nature of inappropriate prescribing by including a list of potentially inappropriate medications (STOPP criteria) and potential prescribing omissions (START criteria) [16]. Complementary to this, the regularly updated American Geriatrics Society’s Beers Criteria is a list of potentially inappropriate medications that older adults should avoid in most situations or in connection with particular health conditions [17]. By reducing inappropriate prescriptions, medication reviews also prevent prescribing cascades—a phenomenon in which an MRP is misinterpreted as a new medical condition, leading to the inadvertent prescription of a new, unnecessary medication to treat it [18].

Implementing effective primary care medication management for home-dwelling older adults with multiple chronic conditions should be a priority to address and prevent adverse health outcomes, avoid hospital admissions, promote and maintain autonomy in their daily lives, and, consequently, help them remain at home [19]. Primary care management requires interprofessional collaboration across different health care and social care providers, organizations, and departments [20]. Indeed, the complexity of dealing with home-dwelling older adults with multiple chronic conditions and needs leads them to be significant users of health services and consult different health care professionals [21]. Besides prescribers—usually general practitioners (GPs) and different specialist physicians—pharmacists, nurses, and other allied health professionals may also be involved in medication management [20]. Collaborative home medication reviews (ie, involving a GP and pharmacist) have demonstrated their effectiveness in preventing, detecting, and resolving MRPs among home-dwelling older adults [20]. Furthermore, adherence to prescribed medication can be improved by pharmacist- and nurse-led interventions [22-25]. However, the number of health care professionals consulted by home-dwelling older adults has been directly associated with fragmented and uncoordinated care [20]. Moreover, different health care professionals may have different treatment preferences, leading to differing prescriptions. Thus, home-dwelling older adults with multiple chronic conditions often receive inefficient and ineffective care [26] or uncoordinated care, even though they are more likely to benefit from it [27]. Failure to coordinate care contributes to MRPs [20]. Older adults who consult ≥3 health care professionals report receiving conflicting advice, making it difficult for them to know whose guidance they should follow. Inadequate explanations about medication result in omissions and incorrect dosages, but they can also lead to anxiety and confusion among older adults [28]. Older adults’ safety could benefit from a more empowering approach, with health care providers sharing information on best practices in medication management and agreeing on responsibilities with older adults and their informal caregivers [29]. The World Health Organization defines empowerment as “a process in which patients understand their role, are given the knowledge and skills by their health care provider to perform a task in an environment that recognizes community and cultural differences and encourages patient participation” [30]. Four components have been reported as being fundamental to the process of patient empowerment: (1) they understand their own role, (2) they acquire sufficient knowledge to engage with their health care provider, (3) they develop skills, and (4) there is a facilitating environment [31]. To the best of our knowledge, these 4 components have never been explored for empowering older adults’ and informal caregivers’ medication management skills and promoting their active engagement in reducing the risk of MRPs.

In addition to health care professionals’ contributions to effective medication management and identifying the risks of MRPs, informal caregivers could help ensure the safe and appropriate use of medication at home by older adults with multiple chronic conditions, notably among those who may also have a cognitive impairment or psychopathological disorder [32-34]. Informal caregivers are defined as any family member, neighbor, or friend who assists a dependent older adult. That assistance, help, care, or physical presence must be regular for ≥6 months, for at least 2 basic or instrumental activities of daily living, or to ensure patient safety [35]. Further interventional studies are required to explore how informal caregivers can be efficiently supported in their roles as medication managers, notably through the use of up-to-date, accurate, and easily understandable medication plans. Primary health care professionals should be assigned the task of cooperating with informal caregivers and integrating their shared decision-making experiences [32-34].

Previous research has shown the effectiveness of interprofessional intervention programs for reducing MRPs. These programs include risk assessments, health education for older adults and their informal caregivers, digital applications, and specialized counseling services or interventions provided by pharmacists and advanced practice nurses [32-34]. These elements should be tested in a standardized, evidence-based, multicomponent, interprofessional intervention program that embeds care in the context of where and how older adults live to prevent the onset or progression of MRPs.

### Objectives

Before conducting a full-scale study with sufficient statistical power, a pilot and feasibility study is critical in the process of developing and testing the intervention. This is “a small-scale test of the methods and procedures to be used on a larger scale” [36]. Thus, our pilot and feasibility study’s goal is not to test hypotheses about our intervention’s impact but to assess the feasibility and acceptability of our approach [37].

### Pilot Study Design

This quasi-experimental, pre-post, multisite pilot and feasibility study will focus on the feasibility components using an open-label design with participants knowing the study’s objectives and relevant information. This will support the development of a standardized, evidence-based, multicomponent, interprofessional intervention program to reduce MRPs.

### The OptiMed Pilot Study’s Framework

**Figure 1:**
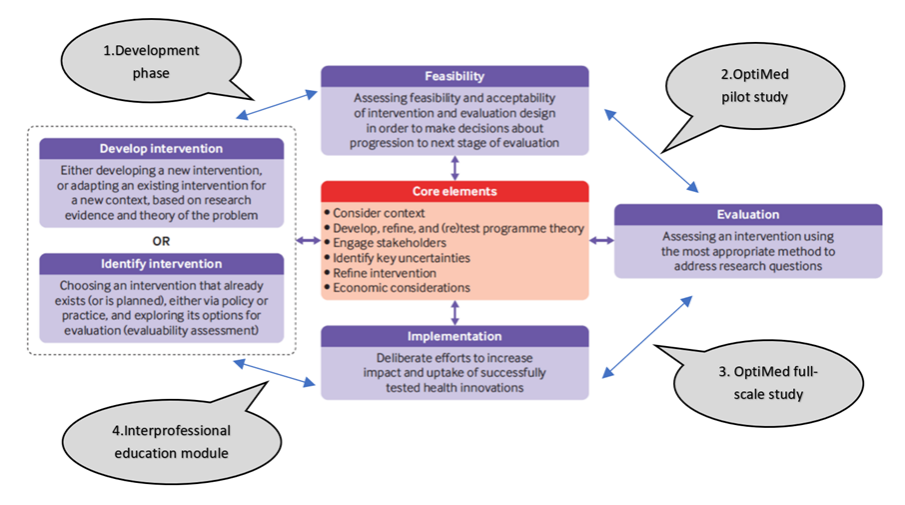
The 4 phases of the pre-post multisite OptiMed study and their relationships with the Medical Research Council’s framework guidelines for complex interventions [38,39].

Intervention studies in primary care are typically complex, multicomponent, interprofessional, and standardized, requiring considerable planning to be conducted successfully [38]. Thus, in accordance with the Medical Research Council’s framework guidelines, we carefully planned the different steps required for the successful execution of this project with sufficient statistical power and the necessary number of participants in both countries (Figure 1) [39]. Designing the OptiMed pilot and feasibility study protocol is part of the development phase, which includes developing all the study’s different components: identifying research gaps and the risks of MRPs, describing relevant concepts, and designing the intervention. Although the OptiMed pilot study (phase 2) will allow us to explore and test the feasibility of conducting the full-scale OptiMed study (phase 3), the latter will assess the effectiveness of the evidence-based, multicomponent, interprofessional medication management intervention program for polymedicated home-dwelling older adults. A comparative analysis of the resources used (costs) and outcomes obtained (effectiveness) will help estimate the health and non–health costs and benefits of this intervention program. As a secondary end point, we also expect to develop and implement a Swiss-Portuguese interprofessional training course about optimizing medication management among polymedicated home-dwelling older adults receiving community-based health care (phase 4).

Furthermore, the following 4 concurrently interacting components that improve the primary care of home-dwelling older adults with multiple chronic conditions—previously identified by Boult and Wieland [40]—will help to operationalize and implement the intervention program: (1) appropriate tools to comprehensively assess the risks associated with MRPs; (2) evidence-based, multicomponent, interprofessional care planning, interventions, and monitoring; (3) the promotion of active engagement in that care by patients and informal caregivers; and (4) coordination between professionals to ensure the best patient care. All 4 concurrent interacting components were tailored to meet the patient’s goals and preferences. In this pilot study, these 4 components will be integrated into a medication management program focused on the context surrounding polypharmacy. Assigning roles and respecting the competencies of each member of the program’s interprofessional team is key to ensuring optimal coordination and a systematic team approach.

## Methods

### Participants, Interventions, and Outcomes

#### Study Setting

The OptiMed pilot study will be conducted in 2 primary health care centers (PHCs): the PHC “Unidade de Saúde Familiar Santiago–Marrazes” in Leiria, Portugal, and another in the French-speaking part of Switzerland.

#### Eligibility Criteria

The research population will include polymedicated home-dwelling adults aged ≥65 years, both men and women, at risk of MRPs and receiving community-based health care. Purposive sampling will recruit older adults taking ≥4 medicines per day and presenting with a risk of an MRP, as assessed using the doMESTIC risk tool, whose psychometric validation is ongoing. The criterion of ≥4 drugs is based on the Delphi study that developed this tool [41]. Older adult participants at risk of MRPs will be integrated into our investigation if their informal caregiver is involved in medication management and they provide informed written consent to participate. On the basis of our clinical experience, being a significant partner in an older adult’s medication management involves at least one of the following activities: accompanying the patient to health consultations, assistance in obtaining medication, collecting or delivering medication, assistance with preparing or taking medication, monitoring effectiveness and side effects, assistance in maintaining the medication schedule, and organizing home care support.

Furthermore, throughout the investigation, each participant will be accompanied by the health care professionals designated as the professional most involved in their medication management. Table 1 presents the inclusion and exclusion criteria for each group of participants.

**Table 1:** Inclusion and exclusion criteria for OptiMed pilot study participants.

Participants		Inclusion criteria	Exclusion criteria
**Study participants**			
	**Polymedicated home-dwelling older adults**		
		Aged ≥65 years for both men and women People with multiple chronic conditions (≥2) Managing at least 4 ore than 90 days [2] (explored during recruitment) At risk of MRPs^a^ identified using the doMESTIC RISK^b^ tool Living alone or with a partner in a rural or urban area Supported by a primary health care center An informal caregiver is involved in medication management Given their written informed consent (Multimedia Appendix 1)	Unable to speak and understand French (Swiss site) or Portuguese (Portuguese site) Moderate or severe dementia criteria (≥10 on the 6-CIT^c^) No risk of MRPs (doMESTIC RISK tool score <5^d^) No informal caregiver
**Collaborating participants**			
	**Informal caregivers**		
		Designated by the older adult as the most significant informal caregiver involved in their medication management Aged ≥18 years Given their written informed consent (Multimedia Appendix 2)	Unable to speak and understand French (Swiss site) or Portuguese (Portuguese site) Moderate or severe dementia criteria (≥10 on the 6-CIT) No risk of MRPs (doMESTIC RISK tool score <5^d^) No informal caregiver
	**Nurse primary care manager**		
		Working for a primary health care center Designated by the older adult as playing a key role in their medication management Given their written informed consent (Multimedia Appendix 3)	Student Apprentice
	**Community pharmacist**		
		Working in a community pharmacy Designated by the older adult as playing a key role in their medication management Given their written informed consent (Multimedia Appendix 3)	Student Apprentice
	**Physician**		
		Older adult’s general practitioner or specialist physician Designated by the older adult as playing a key role in medication management Given their written informed consent (Multimedia Appendix 3)	Student Apprentice

^a^MRP: medication-related problem.

^b^doMESTIC RISK: Medication Safety in Home Care.

^c^6-CIT: 6-item Cognitive Impairment Test.

^d^As assessed by the research team.

#### Primary and Secondary Feasibility and Acceptability Outcomes

The primary objective of this pilot study is to explore the feasibility and acceptability of an evidence-based, multicomponent, interprofessional intervention program with the support of informal caregivers to decrease MRPs among polymedicated home-dwelling older adults with multiple chronic conditions. As per Eldridge et al [42], a feasibility study “asks whether something can be done, should we proceed with it, and if so, how.” Therefore, in line with the Medical Research Council’s framework guidance [39], our study will gather information about the feasibility of recruitment, participant retention, intervention adherence (adherence to instructions by participants), intervention fidelity, and intervention dose [36,43] (Textbox 1 [44]). The quantitative cutoff point for our feasibility outcomes will be successful with at least 70% of participants [45]. Given the older adult participants’ advanced age and chronic health conditions, the number of withdrawals for health reasons may be higher than in other studies. Acceptability will be assessed by measuring participants’ satisfaction with each assessment instrument, home visits, and the targeted education plan. The secondary objective is to test the different data collection tools planned for use in the full study.

**Outcomes and measurements of the OptiMed pilot study’s feasibility and acceptability, based on the United States National Center for Complementary and Integrative Research recommendations [44].**

- Measurements of feasibility
  The target population is recruited: the number screened per month, the number enrolled per month, and the average time between screening and enrollment
  Study participants are retained: treatment-specific retention rates for study measurements and reasons for dropouts
  Study participants adhere to their instructions: treatment-specific rates of adherence to the study protocol (in-person session attendance, homework, home sessions, etc) and treatment-specific measurements of competencies
  The medication review is successful: treatment-specific fidelity rates, time invested, number of discrepancies between professional sources, number of discrepancies between patient-reported medication use and professional sources, number of questions, number of items needing clarification, number of recommendations made by the pharmacist, and number of pharmacist’s recommendations adopted by the physician
  The credibility of each component of the interprofessional intervention program is assessed as positive: ratings for treatment-specific expectations of benefit
  The relationship between the dose of nursing [46] and response to the program is measured: amount, frequency, intensity, and duration
  Study displays clinical relevance: how participants perceive the intervention program, using patient-reported outcome measures [47]. Identify what is relevant for participants (such as quality of life or remaining at home). Enabling a choice of which outcomes from the pilot study will go into the intervention
- Measurements of acceptability
  Participants are satisfied with each assessment instrument, home visits, and the targeted education plan: acceptability ratings are calculated using quantitative assessments, reasons for dropouts, and treatment-specific preference ratings (pre- and postintervention)
  Assessments are not considered too burdensome: the proportion of planned assessments that are completed; duration of assessment visits; reasons for dropouts; ease of use of the doMESTIC RISK tool; ease of use of the structured medication review template

According to the Consensus-based Standards for the Selection of Health Measurement Instruments (COSMIN) recommendations [48], if the pilot study is conclusive, then the full-scale OptiMed study will adopt the standardized Core Outcome Set for its clinical trials of a medication review for multimorbid older adult patients with polypharmacy, as previously reported by Beuscart et al [49] in 2018 (Multimedia Appendix 4).

#### Study Intervention

Completing the intervention program with 1 participant requires 5 weeks. After the older adult participant has been recruited via their nurse primary care manager working for their PHC, the intervention’s first component involves their baseline assessment at a meeting conducted by a research nurse (t0). The research is explained to the older participant in detail, both orally and in writing, and the consent form is signed (Multimedia Appendix 1). After this, the older participant is asked to answer some sociodemographic and health-related questions and provide a complete list of their current medication prescription. The research nurse completes a questionnaire on the risk of MRPs—doMESTIC risk questionnaire (Multimedia Appendix 5). A first meeting was also conducted individually with the informal caregiver (who will also answer some sociodemographic and health-related questions and describe their role in medication management; Multimedia Appendix 6). The older adult participant is asked to identify the health care professionals (GP or specialist, nurse primary care manager, or community pharmacist) most involved in their medication management.

The second component involves a review of the prescribed medications in week 1 (t1), including a medication reconciliation and a structured medication analysis by the study’s partnering pharmacist to identify drugs with a high risk of MRPs among older adults. This will use the STOPP and START criteria, and undertreated indications or missed therapies will use the START criteria [16]. The older adult participant’s physician will be invited to participate in the review of prescribed medications in collaboration with the study’s partnering pharmacist. They will discuss validated, evidence-based, and internationally recognized guidelines to reconsider beneficial or nonbeneficial therapies or to simplify and focus on specific care goals and adjust medications to be consistent with the guidelines. If the older adult participant’s physician adopts none of the proposed changes, despite the medication review (t1) highlighting their relevance, the research team will inform the older adult participant of their physician’s decision and invite them to proceed with the study.

On the basis of the baseline assessment (t0), the research nurse will use a joint consultation in week 2 to explore the needs and care goals of the older adult participant and their informal caregiver to reduce the risk of MRPs (t2). In collaboration with the designated health care professionals, the research nurse will then design a targeted patient-centered education plan to empower older adult participants’ medication management and reduce the risk of MRPs (t2). This is based on a four-step patient empowerment process: (1) the patient understands their role, (2) the patient acquires sufficient knowledge to engage with their health care professionals, (3) the patient improves their skills, and (4) there is a facilitating environment [31]. Each older adult participant’s plan will be unique and consider their preferences, medication literacy, and treatment adherence.

During weeks 3 and 4 (t3), the research nurse will help the older adult participant and their informal caregiver to implement their targeted education plan (t2) aimed at empowering their medication management and promoting their active engagement in reducing the risks of MRPs. Two joint consultations will be organized among the older adult participant, their informal caregiver, and the research nurse (once each week). During planned home visits, the nurse will periodically evaluate the older adult participant’s medication status (primary and secondary outcomes) and promote communication between different professional and nonprofessional actors involved in medication management.

The research nurse will complete a final assessment (t4) of each older adult participant’s risks of MRPs in week 5 (35-40 days after t0) in collaboration with the study’s partnering pharmacist. The occurrence of MRPs and medication-related hospital admissions will be investigated. Finally, the health care professionals designated by the older adult participant will assess the acceptability of the interprofessional intervention program aimed at reducing MRPs by completing a final questionnaire (Multimedia Appendix 7). The pilot study will explore possible biases so that they can be correctly addressed in the full study (Multimedia Appendix 8) [36,38].

As already described, the medication management intervention program guiding OptiMed’s pilot study comprises 4 components based on concurrently interacting processes previously identified by Boult et al [40] (Multimedia Appendix 9).

The schedule of enrollment, interventions, and assessments is presented in Table 2. The pilot study will be conducted over 12 months, including the recruitment phase (Multimedia Appendix 10).

**Table 2:** OptiMed schedule of enrollment, interventions, and assessments based on Standard Protocol Items: Recommendations for Interventional Trials [50].

Time point						Study period																					
						Enrollment			Allocation				Postallocation													Final end point	
						–t_1_			t0				t1				t2				t3 Wk3, Wk4, Wk5				t4		
**Enrollment**																											
	Eligibility screening					✓																					
	Informed consent					✓																					
	Other procedures (to complete)																										
	Allocation					Not applicable in the pilot study			Not applicable in the pilot study				Not applicable in the pilot study				Not applicable in the pilot study				Not applicable in the pilot study				Not applicable in the pilot study		
**Interventions**																											
	Medication review process examining the risks of MRPs^a^																										
	Targeted education plan to empower older adults’ and informal caregivers’ medication management to reduce the risks of MRPs																										
	Implementing the targeted plan to reduce the risks of MRPs																										
**Assessments**										✓																	
	**Baseline variables**																										
		Older adult participants’ sociodemographic data health status, and risks of MRPs																									
		Informal caregivers’ sociodemographic data						✓																			
		Health care professionals’ sociodemographic and professional data						✓																			
	**Final assessment of older adult participants’ risks of MRPs**																										✓
		**Medication reconciliation^b^**																									
			Number of discrepancies								✓																
			Number of clarifications needed								✓																
		**Medication analysis^b^**																									
			Overuse								✓												✓				
			Underuse								✓												✓				
			Potentially inappropriate medications								✓												✓				
			Clinically significant drug-drug interactions								✓												✓				
			Monitoring requirements								✓												✓				
		**Adverse events^a^**																									
			Medication-related hospital admissions																				✓				
			MRPs																				✓				
		Patient-reported outcomes^c^ (will be defined by the pilot study)																					✓				
		**Feasibility outcomes**																									
			Target population recruitment																				✓				
			Retaining study participants																				✓				
			Participants’ adherence to what they are asked to do																				✓				
			Medication reconciliation																				✓				
			The burden of assessments																				✓				
			Acceptability of each intervention																				✓				
			The credibility of each intervention																				✓				
			Relationship between the dose of nursing [46] and response to the program																				✓				

^a^MRP: medication-related problem.

^b^Primary outcomes.

^c^Secondary outcomes.

#### Sample Size and Recruitment

On the basis of the study by Hogget al [51], we planned to recruit 30 older adult participants (15 men and 15 women), 30 informal caregivers, and 30 health care professionals in collaboration with 2 PHCs: the Unidade de Saúde Familiar Santiago–Marrazes in Leiria, Portugal, and another in the French-speaking part of Switzerland. Nurse primary care managers partnering with the project will briefly explain the study to polymedicated home-dwelling older adults who meet the inclusion criteria. Eligible participants will be asked for permission to provide their names to the researchers, and a research team member will telephone them to ask for their agreement to participate in the study. If they agree, the first meeting with a research team member will be organized at the older adult participant’s home or the PHC in the following few days. Given the 2 countries’ different primary health care practices, eligible participants in Switzerland will be met at home, whereas participants in Portugal will be met at the PHC. Consenting older adult participants will be asked to designate the most significant informal caregiver, aged ≥18 years, involved in their medication management, and the research team will invite them to participate in the study. The older adult participants will be asked to designate the health care professionals (GPs or specialist physicians, nurse primary care managers, or community pharmacists) who are most prominently involved in their medication management. During the first meeting, the research nurse will explain the study’s nature, purpose, and procedures to the older adult participant, along with its expected duration, potential risks and benefits, and any discomfort it may entail. Each older adult participant will be informed that participation in the study is voluntary, that they may withdraw from it at any time, and that the withdrawal will not affect their subsequent medical assistance and treatment.

### Data Collection, Management, and Analysis

#### Data Collection Methods

Data on older adult participants’ health status will be collected using the 6-item Cognitive Impairment Test (6-CIT), Tilburg Frailty Indicator (TFI), and the diagnosis list based on the 11th revision of the International Statistical Classification of Diseases and Related Health Problems (ICD-11). Current medication, previous MRPs, and the doMESTIC RISK tool will provide data on the risks of future MRPs and will be collected, when possible, from routinely collected PHC data or directly from older adult participants and their informal caregivers.

Sociodemographic data and information about informal caregivers’ contributions to their relatives’ medication management will be collected. We will collect sociodemographic and professional data from health care professionals (GPs, nurse primary care managers, and community pharmacists), along with information about medication monitoring and follow-up, how they teach and train older adults and informal caregivers on medication management, how they assess their cognitive capacity to manage medication, stakeholder collaboration, and medication management.

The study will also collect participants’ opinions on the acceptability of each intervention, reasons for dropout, and treatment-specific preference ratings (before and after the intervention).

Except for the doMESTIC risk tool, whose psychometric validation is ongoing, all other instruments in the study have demonstrated their reliability and validity in previous studies. Table 3 describes the measurements taken, the instruments used for data collection, and their respective purposes. To promote data quality, the nurses and pharmacists partnering with the study will undergo two 1-hour training sessions on assessment.

**Table 3:** Measurements and instruments used for data collection.

Participants			Measurements and instruments		Purpose
**Study participants**					
	**Home-dwelling older adults**				
		Sociodemographic data (baseline assessment, t0; Multimedia Appendix 5)		Describe older adult participants’ profiles (birthdate, gender household size, number of hospitalizations, and the date of the last hospitalization, etc)	
		Six-item Cognitive Impairment Test (t0; Multimedia Appendix 5)		Perform brief cognitive screening recommended for use in primary care settings [52]	
		TFI^a^ (self-administered or at t0; Multimedia Appendix 5)		Assess multidimensional frailty among older adult participants via a self-administered questionnaire [53]	
		11th revision of the ICD-11^b^ diagnosis list (t0)		Describe older adult participants’ morbidities based on the ICD-11 [54]. The list is useful when doing a medication analysis and evaluating prescription adequacy	
		Current medication lists (t0)		Perform medication analysis (overuse, underuse, potentially inappropriate medications, and clinically significant drug-drug interactions) and medication reconciliation (number of discrepancies and clarifications needed)	
		Previous MRPs^c^ (t0)		Describe the history of past MRPs	
		doMESTIC RISK Tool (t0 and final assessment, t4)		Identify older adult participants at a high risk of MRPs [41]	
		Acceptability of the intervention [55] (t4; Multimedia Appendix 5)		Collect older adult participants’ opinions on the acceptability of the medication management intervention program	
**Collaborating participants**					
	**Informal caregivers**				
		Sociodemographic data (t0; Multimedia Appendix 6)		Describe informal caregivers’ profiles and their relationships with older adult participants	
		Involvement in medication management (t0; Multimedia Appendix 6)		Describe types, frequencies, and intensities of informal caregivers’ support activities	
		Acceptability of the intervention [55] (t4; Multimedia Appendix 6)		Collect informal caregivers’ opinions on the acceptability of the medication management intervention program	
	**Health care professionals (nurse primary care manager, community pharmacist, and physician)**				
		Sociodemographic and professional data (t0; Multimedia Appendix 7)		Describe professional caregivers’ profiles and baseline involvement in medication management	
		Acceptability of the intervention [55] (t4; Multimedia Appendix 7)		Collect professional caregivers’ opinions on the acceptability of the medication management intervention program	

^a^TFI: Tilburg Frailty Indicator.

^b^ICD: International Statistical Classification of Diseases and Related Health Problems.

^c^MRP: medication-related problem.

#### Study Instruments

##### 6-Item Cognitive Impairment Test

6-CIT was selected for the cognitive screening of older adult participants because it is brief, simple to apply, and requires no special training for administration. The 6-Item Orientation-Memory-Concentration Test was originally developed by Katzman et al [56] by shortening the Mental Status Test by Blessed et al [57]. The 6-CIT involves 3 temporal orientation tests (year, month, and time), 2 attention tests (counting backward from 20 to 1, reciting the months of the year in reverse), and 1 short-term memory test (a 5-item address) [52]. Scored out of 28, higher scores indicate greater impairment. The 6-CIT has been used in a broad range of settings, including screening for dementia in primary care, cognitive screening in acute care, large population-based studies, and Alzheimer disease studies [52]. In primary care, the English version’s internal consistency (Cronbach α), stability over time (Pearson *r*), and agreement between successive tests (Cohen κ) reached values of .58, 0.62, and 0.45, respectively [58]. Sensitivity and specificity reached values of 0.49 and 0.92 at the 7/8 cutoff and 0.32 and 0.98 at the 10/11 cutoff, respectively [58]. The Portuguese version of the 6-CIT (6-CIT-P) presented a high test-retest reliability coefficient (*r*=0.95, *P*<.001; n=54), indicating good temporal stability [59]. 6-CIT-P has also shown strong internal consistency (Cronbach α=.88), and the corrected item-total correlations ranged between 0.32 and 0.90, representing a moderate-to-strong correlation between the items and total score. The 6-CIT-P and mini-mental state examination scores were strongly negatively correlated (*r*=−0.90) because of the polarity of the tests, indicating an acceptable convergent validity. The Global 6-CIT showed good sensitivity and specificity (82.78% and 84.84%, respectively) compared with the mini-mental state examination [59]. The French version is currently undergoing a validation study.

##### Tilburg Frailty Indicator

As polypharmacy has been associated with a higher incidence of frailty [60,61], the TFI will be used to assess multidimensional frailty among home-dwelling older adults [53]. Unlike other frailty assessment instruments, TFI does not focus exclusively on the physical dimension of frailty and does not include disability and diseases in its assessment. This contributes to a holistic perspective of human functioning in the community (including the physical, psychological, and social domains). The TFI consists of 2 parts: part A contains 10 questions on the determinants of frailty and multi-morbidity, whereas part B uses 15 questions to examine 3 domains of frailty (physical, psychological, and social) [53]. Each question is scored as 1 or 0. A total score of ≥5 suggests that a person is frail and the greater the score, the greater the degree of frailty is considered significant. The original English version’s internal coherence demonstrated a satisfactory Cronbach α coefficient of .73 [62]. Construct validity between the different domains revealed significant Pearson correlation coefficients (*P*<.05): *r*=0.42 between the physical and psychological domains; *r*=0.19 between the physical and social domains; and *r*=0.18 between the psychological and social domains. Convergent and divergent validities were considered to be good. The TFI demonstrated good temporal-fidelity stability, with a frailty score of 0.90 after 2 weeks and 0.79 after 1 year [62]. The Portuguese version of the TFI showed good internal consistency (KR-20=0.78) and test-retest reliability (*r*=0.91), with Cohen κ coefficients showing substantial agreement for most items [63]. The French version is currently under validation.

##### doMESTIC RISK Tool

The doMESTIC RISK tool is used to identify patients at high risk of MRPs [41]. It is based on home-care–specific risk factors for medication safety as derived from the literature. Experts prioritized the tool’s suggested items using a 2-round Delphi study. The final tool was statistically validated using a systematic medication review involving 150 home care patients. The tool’s subsequent weighting improved the model’s overall quality and resulted in a final doMESTIC RISK tool with 10 risk factors. The tool’s strengths are its specific focus on the home care setting and the interprofessional input necessary for its completion [41]. French and Portuguese versions of the original tool will be used. The reliability, validity, and responsiveness of the doMESTIC RISK tool are being measured based on a quantitative analysis of field tests (factor analysis and item response theory) [48,64]. In this pilot study, an older adult’s risk of MRPs and whether the medication management intervention program contributes to decreasing it will be assessed by 2 nurse research assistants in collaboration with 2 pharmacists using the doMESTIC RISK tool at the baseline assessment (t0) and the final assessment (t4).

#### Participant Retention and Completing Follow-up

On the basis of the best practice guidance [65], the following strategies will be used to promote the retention of older adult participants and informal caregivers and to complete follow-up:

- Involving informal caregivers.
- Allowing rest between measurements and interviews, as needed.
- Meeting older adult participants at home during visits by the PHC’s home care services (Switzerland).
- Arranging transport to the meeting location or reimbursing transportation costs (Portugal).
- Keeping meetings short (<1 hour).
- Providing opportunities to build social support through organized educational sessions within the targeted plan.
- Keeping in touch by scheduling appointments in advance and sending reminders to both the older adult participant and informal caregiver.
- Providing regular feedback to study participants.

Participants will be able to withdraw their consent to participate in the study at any time, without having to justify themselves. Participants who discontinue, drop out, or deviate from the intervention program will be briefly interviewed to learn when and why they did so, and an intention-to-treat analysis will be conducted. In case of withdrawal from the study, the data collected will be anonymized using secuTrial search software and stored on secure servers at the School of Health Sciences, HES-SO Valais-Wallis, or at ciTechCare, Polytechnic of Leiria.

#### Data Management

All data will be entered into the secuTrial electronic data capture system, either at the School of Health Sciences, HES-SO Valais-Wallis, or at ciTechCare, Polytechnic of Leiria. Original study forms will be scanned, stored electronically with limited access, and kept on file at the HES-SO and ciTechCare sites. Data entry screens will resemble the paper forms approved by the ethics committees. Data consistency will be ensured through various mechanisms, including referential data rules, valid values, range checks, and consistency checks against data already stored in the database (longitudinal checks). Checks will be applied when data are entered into a specific field and before they are committed permanently to the database. Modifications to the data in the database will be documented via an audit trail. Data in the secuTrial database will be retrievable through data entry applications. The types of activities that individual users may undertake will be regulated by the privileges associated with their user identification code and password.

The 2-country roll-out will allow greater standardization of the evidence-based, multicomponent intervention program for a future, full-scale, quasi-experimental, pre-post study. However, raw data will not be shared between countries, and the results obtained will be discussed only after being analyzed separately in each country. A joint publication is planned using aggregated data from the pilot study results.

#### Statistical Methods

The pilot study will compute descriptive statistics as distributions and central tendencies, as well as inferential statistics, to provide power analyses for the pilot study’s proposed sample size. The pilot study’s results will help provide a power analysis of the sample needed for a fully powered pre-post study. Analyses will be conducted in collaboration with a biostatistician using an up-to-date version of the SPSS software.

#### Handling Missing Data and Dropouts

We do not expect any missing data, as the protocol is limited and properly adapted to the potential participants’ characteristics. The OptiMed pilot study will consider missing data and dropout rates as significant results for organizing recruitment, intervention adherence, acceptability, and subject retention in the full-scale study. Nevertheless, to fully evaluate the different components of the pilot study, we will replace dropouts with new participants until 30 of them have completed the study. Given the intervention program’s 5-week duration and the older adult participants’ multiple chronic health conditions (as they are all polymedicated), it is difficult to anticipate how many will complete the intervention and how many may interrupt it for health or availability reasons. This is also one of the rationales for conducting a pilot feasibility study before conducting a full-scale study.

### Monitoring

#### Data Monitoring

secuTrial will help in monitoring both the pilot study and the full-scale study. Data monitoring will inform us of aspects of the study’s conduct, such as ongoing recruitment, and help identify any need for adjustment.

Lausanne University Hospital’s Clinical Trial Unit (CHUV-CTU) and Leiria’s Regional Health Administration (“Administração Regional de Saúde” – ARS) will check the electronic case report forms (eCRFs) for consistency and completeness throughout the study. If an issue is detected, the monitor will open a query in the secuTrial software, and the investigator will be able to answer and correct the data in that software as needed. An overall data consistency check will be performed by the study monitor and the designated data manager. Any actions required to fix the data issues will be reported in the eCRFs. A final export of the data set will enable us to completely remove participants’ identities, and the database will be locked.

#### Risk-Benefit Assessment

The research team will not add or change any medication. However, based on a medication review (made following validated, evidence-based, and internationally recognized guidelines), the study’s partnering pharmacist may provide suggestions to older adult participants’ GPs or specialist physicians. Thus, by participating in the project, older adult participants may be exposed to minor risks, such as a temporary alteration of their usual condition, if their physician modifies their treatment after discussion with the study’s partnering pharmacist to avoid MRPs.

Medication management will be performed using validated, internationally recognized, and evidence-based guidelines for older adults. The medication management intervention program proposed to the older adult participants and their informal caregivers will be tailored (in intensity and duration) to each individual and will consider their health and medication literacy. This is important to avoid a lack of follow-up, particularly among those with a possible undiagnosed mild cognitive impairment. Older adult participants, informal caregivers, and prescribers should benefit from a more empowering approach to medication management.

#### Insurance

In case of any study-related damage or injury, the School of Health Sciences, HES-SO Valais-Wallis, and ciTechCare, Polytechnic of Leiria, may be liable for compensation, except for claims that arise from misconduct or gross negligence.

### Auditing

#### Overview

CHUV-CTU and ARS will regularly monitor data quality and completeness. They will audit source documents in Switzerland and Portugal, respectively, with at least 1 research site monitoring visit every 6 months in each country throughout the study. The primary objectives of this study are education, support, and problem solving. The monitors will discuss the protocol in detail and identify and clarify any areas of weakness. The investigators will practice entering data so that the study monitors are assured of their proficiency in all aspects of data entry, query response, and communication with the CHUV-CTU and ARS. The monitors will audit the overall data quality and completeness, examine source documents and interview investigators, and confirm that the research site has complied with the protocol’s requirements. The monitors will verify that all adverse events were correctly documented and consistent with the protocol’s definition. They will review source documents (ICD-11 diagnosis list, current medication lists, and associated reports from PHCs) as needed to determine whether the data reported in secuTrial are complete and accurate. They will confirm that the regulatory binder is complete and that all associated documents are up to date. The regulatory binder should include the protocol, informed consent forms, ethics approval from both countries, and investigator agreements.

If a problem is identified during a visit (eg, poor communication with the CHUV-CTU and ARS, inadequate or insufficient staff to conduct the study, and missing study documents), the monitors will assist the research site in resolving its issues. Visits and electronic monitoring will focus on source document reviews and confirmation of adverse events. Monitors will verify the following variables for all study participants: date of birth, sex, signed informed consent, eligibility criteria, treatment assignment, adverse events, and end point. All parties involved will keep participants’ data strictly confidential.

#### Ethics Approval

Ethics approval was obtained from the Human Research Ethics Committee of the Canton of Vaud (Commission cantonale d'éthique de la recherche sur l'être humain), Switzerland (2021-01843), and Leiria’s ARS, Portugal (80-2021).

#### Protocol Amendments

Any substantial changes made to the study setup or organization, protocol, or relevant study documents will be submitted to the CER-VD and ARS for approval before implementation. In an emergency, deviations from the protocol may be made to protect human subjects’ rights, safety, or well-being without the Ethics Committee’s prior approval. Such deviations will be promptly documented and reported to the CER-VD and ARS.

#### Consent to Participate

All study participants will be provided with a participant information sheet and a consent form describing the study and providing sufficient information for them to make an informed decision about participation (Multimedia Appendices 4, 5, and 6). Participants will have up to 2 weeks to decide whether to participate in the study. Formal consent will be obtained using an approved consent form before the participant is sent for any study procedure. The investigator will sign and date the informed consent form simultaneously with the participant, 1 copy will be given to the study participant and the other will be retained in a secure location as part of the study records. Participants will also be requested to provide written informed consent for reusing the data in an encoded form.

Before the pilot study, the intervention program will be pretested on 3 to 4 study participants to evaluate how long, intense, and tiring it is. Participants’ autonomy will be respected, and study participation will be voluntary. They will be able to refuse further participation or drop out of the study at any time. However, once collected, data will not be deleted. Throughout the process, the research team will tell the truth and ensure that it misleads neither its research partners nor the participants.

Older adult participants, their informal caregivers, and collaborating health care professionals will receive no compensation for their involvement in this research project. However, the study participants will benefit from the intervention program and provide superior support for medication management.

#### Confidentiality and Access to Data

Participant data will be handled with the utmost discretion and will only be accessible to authorized personnel who require that data to fulfill their duties within the scope of the study. In the eCRFs and other study-specific documents, participants will only be identified using their unique participant numbers. Study participants will be coded as follows: OPTIMED-01, OPTIMED-02, etc. Any links between that code and the participant’s real identity will be protected by secuTrial, and only the research team will have access to the data collected. The types of activities that individual users may undertake will be regulated by the privileges associated with their user identification code and password. The original study forms will be scanned, stored electronically with limited access, and kept on a secure server at the School of Health Sciences, HES-SO Valais-Wallis, and at ciTechCare, Polytechnic of Leiria, for 20 years after the completion of the study.

#### Dissemination Policy

Knowledge transfer activities will promote this pilot study at professional and scientific conferences for primary health care professionals, within hospital care units, and to informal caregivers’, patients’, and older adults’ associations. The findings of the pilot study will be submitted to a peer-reviewed journal.

## Results

This paper describes the development of a pre-post, multisite pilot, and feasibility study to explore the feasibility and acceptability of an interprofessional intervention program to decrease MRPs among polymedicated, home-dwelling older adults with multiple chronic conditions. We expect our findings to provide sufficient methodological evidence regarding the rationale, design, and planning of the intervention to warrant a full-scale study. This methodological evidence will include approaches to recruitment and obtaining consent, ensuring adherence to each phase of the intervention program, and easing the burden of assessments. Although this pilot and feasibility study did not have sufficient statistical power to provide estimates of effect size, we expect that our findings will provide useful data to help define the final components of the full-scale study and research strategies.

## Discussion

Designing, constructing, and exploring the effectiveness of an intervention program to reduce the risk of MRPs among home-dwelling older adults, in collaboration with all the different actors involved in their medication management, is an emerging but underinvestigated issue. Current knowledge in this area was developed in academic settings and acute and long-term care facilities; however, little is known about this topic in home care settings. Regarding the prevention of MRPs, research to date has been unable to simultaneously integrate the roles and perceptions of polymedicated home-dwelling older adults with multiple chronic conditions, their informal caregivers, and their health care professionals [66]. To close these gaps, our interprofessional intervention has been coconstructed with older adults and their informal caregivers and could contribute significantly to preventing hospital admissions, rehospitalizations, institutionalization in long-term care facilities, and early death. This could also limit the economic impact of MRPs on the already stretched health care systems [67]. The OptiMed study is a tailored intervention program based on a clear allocation of roles to reduce the risk of MRPs among home-dwelling older adults. This medication management program is underpinned by a complex intervention framework that includes development, implementation, and sustainability milestones [39]. It also considers the 4-fold aim of enhancing the patient’s experience, improving population health, reducing costs, and improving health care professionals’ working lives [68].

## Appendix

Multimedia Appendix 1 Older adults’ consent form (in French).

Multimedia Appendix 2 Informal caregivers’ consent form (in French).

Multimedia Appendix 3 Health care professionals’ consent form (in French).

Multimedia Appendix 4 Outcomes OptiMed full-scale study.

Multimedia Appendix 5 Older adults’ questionnaire (in French).

Multimedia Appendix 6 Informal caregivers’ questionnaire (in French).

Multimedia Appendix 7 Health care professionals’ questionnaire (in French).

Multimedia Appendix 8 Feasibility questionnaire.

Multimedia Appendix 9 Overview of the OptiMed pre-post and multisite pilot and feasibility study.

Multimedia Appendix 10 Timetable for the OptiMed pilot study.

## Abbreviations

6-CIT: 6-item Cognitive Impairment Test
ARS: Leiria’s Regional Health Administration
CHUV-CTU: Lausanne University Hospital’s Clinical Trial Unit
eCRF: electronic case report form
GP: general practitioner
MRP: medication-related problem
PHC: primary health care center
START: Screening Tool to Alert to Right Treatment
STOPP: Screening Tool of Older Persons’ Prescriptions
TFI: Tilburg Frailty Indicator

## Data Availability

The data sets used and analyzed during this study are available from the corresponding author upon reasonable request.
