# Supplementary material for: An Intervention Program to Reduce Medication-Related Problems Among Polymedicated Home-Dwelling Older Adults (OptiMed): Protocol for a Pre-Post, Multisite, Pilot, and Feasibility Study
Source: JMIR Res Protoc. 2023 Jan 25;12:e39130. doi: 10.2196/39130 (PMC9909524; doi:10.2196/39130)
Supplement: Multimedia Appendix 3 [file resprot_v12i1e39130_app3.docx]

Demande de participation à un projet de recherche médical :

**Optimisation de la gestion médicamenteuse pour des personnes âgées de 65 ans et plus polymédiquées vivant à domicile : l’étude pilote OptiMed**

Madame, Monsieur

Nous vous proposons de participer à notre projet de recherche.

Votre participation est entièrement libre. Toutes les données collectées dans le cadre de ce projet sont soumises à des règles strictes en matière de protection des données.

Le projet de recherche est mené par la Haute École de Santé, de la HES-SO Valais / Wallis. Nous vous en communiquerons les résultats si vous le souhaitez.

Lors d’un entretien, nous vous présenterons les éléments essentiels et répondrons à vos questions. Pour vous proposer d’ores et déjà un aperçu du projet, voici les points clés à retenir. Vous trouverez à la suite des informations complémentaires plus détaillées.

Pourquoi menons-nous ce projet de recherche ?

- En présence de polymédication, on devrait favoriser l’autonomie des personnes âgées de 65 ans et plus et de leurs proches aidants en matière de gestion des médicaments à domicile, dans le but d’éviter des problèmes liés à la médication.
- Notre projet de recherche vise à déterminer si un programme d'intervention pour une gestion optimale de la médication à domicile, mené par différents professionnels de la santé et soutenu par des proches aidants, est faisable et acceptable pour les participants.

Que dois-je faire si j’accepte de participer ? – Que se passe-t-il pour moi en cas de participation ?

- Forme de la participation : Si vous acceptez de participer à notre projet, vous serez amené·e à rencontrer une fois un membre de l’équipe de recherche. Vous aurez l’occasion de répondre à un questionnaire quant à votre rôle dans la gestion médicamenteuse du·de la participant·e âgé·e de 65 ans et plus et de participer au développement d’un plan d'éducation pour promouvoir l’engagement actif du·de la participant·e âgé·e et proche aidant·e dans la réduction des risques d’évènements indésirables médicamenteux.
- Déroulement pour les participant e s : Si vous participez au projet, vous n’aurez aucune obligation. Lors de la rencontre prévue, vous pourrez tout à-fait dire à l’équipe de recherche que vous ne souhaitez plus participer à l’étude sans devoir vous justifier.

Quels sont les bénéfices et les risques liés à la participation au projet ?

Bénéfices

- Si vous participez à ce projet, vous favoriserez une meilleure compréhension des enjeux liés à la gestion médicamenteuse à domicile. Vous contribuerez à l’élaboration d’un programme interprofessionnel visant à réduire le risque d’évènements médicamenteux indésirables, en promouvant l’engagement actif des personnes âgées et proches aidants dans la gestion du traitement médicamenteux.

Risques et contraintes

- Aucun risque ou contrainte n’est prévu en participant à cette étude.
- Aucune dépense ou frais ne seront occasionnés par votre participation.

En apposant votre signature à la fin du document, vous certifiez en avoir compris le contenu et consentir librement à prendre part au projet.

**Information détaillée**

# **Objectif du projet et sélection des participant·e·s**

Dans cette feuille d’information, notre projet de recherche est aussi simplement désigné par le terme *projet*. Si vous acceptez d’y prendre part, vous êtes *un participant/une participante au projet*.

Ce projet doit nous permettre d’examiner si un programme d'intervention pour une gestion optimale de la médication à domicile, mené par différents professionnels de la santé et soutenu par des proches aidants, est faisable et acceptable pour les participants.

Nous vous sollicitons car la participation est ouverte aux professionnels de la santé (médecin traitant ou spécialiste, infirmière en soins à domicile et/ou pharmacien) de personnes âgées de 65 ans et plus, atteintes de deux maladies chroniques ou plus et ayant au moins quatre médicaments prescrits par jour, vivant seul ou accompagné.e, dans des régions rurales ou urbaines.

# **Informations générales sur le projet**

Une gestion optimale des médicaments est l'une des conditions préalables essentielles pour que les personnes âgées de 65 ans et plus polymédiquées vivant à domicile puissent rester chez elles et préserver leur qualité de vie et leur autonomie.

Nous savons encore peu de choses sur comment favoriser l’autonomisation et le renforcement des compétences des personnes âgées de 65 ans et plus et de leurs proches aidants en matière de gestion des médicaments à domicile. Nous souhaitons donc promouvoir leur engagement actif dans la réduction du risque de problèmes médicamenteux.

Quatorze personnes âgées de 65 ans et plus seront recrutées. Un total de 42 participant·e·s (14 personnes âgées de 65 ans et plus, 14 proches aidants et, au moins, 14 professionnels de la santé) seront donc recruté·e·s tout au long du projet.

L’infirmier.ère des soins à domicile référent·e de la personne âgée de 65 ans et plus, en qualité de partenaire du projet, vous expliquera brièvement la présente recherche. Il vous sera demandé de donner votre accord pour que votre nom soit transmis aux chercheurs. Par la suite, l’équipe de recherche prendra contact avec vous par téléphone afin de vous demander formellement votre accord pour participer à cette recherche.

En tant que participant·e, vous serez amené·e à rencontrer une fois un membre de l’équipe de recherche. Vous aurez l’occasion de répondre à un questionnaire quant à votre rôle dans la gestion médicamenteuse du·de la participant·e âgé·e de 65 ans et plus et de participer au développement d’un plan d'éducation pour promouvoir l’engagement actif du·de la participant·e âgé·e et proche aidant·e dans la réduction des risques d’évènements indésirables médicamenteux.

Ce projet se déroule en Valais, en Suisse et, de manière indépendante, à Leiria, au Portugal. Dans chaque pays seront recrutés 14 personnes âgées de 65 ans et plus, ainsi que leurs proches aidants et leurs professionnels de la santé. Les données des participants ne seront jamais échangées entre les deux pays.

Ce projet est réalisé dans le respect des prescriptions de la législation suisse. Nous suivons en outre l’ensemble des directives reconnues au niveau international. La commission d’éthique compétente a examiné et autorisé ce projet.

# **Déroulement du projet**

L’étude se déroule en cinq étapes distinctes :

Si vous acceptez de participer, la **première étape (semaine 0)** consiste en une rencontre d’approximativement 45 minutes menée par un·e infirmier·ère membre de l’équipe de recherche. La recherche vous sera expliquée en détail, oralement et par écrit. L’infirmier·ère membre de l’équipe de recherche vous proposera de signer la déclaration de consentement qui se trouve à la fin de ce document. A la suite de cela, l’infirmier·ère membre de l’équipe de recherche vous demandera de répondre à quelques questions sociodémographiques. Un questionnaire sur votre contribution à la gestion médicamenteuse du·de la participant·e âgé·e sera complété.

La **deuxième étape (semaine 1)** consiste pour le·la pharmacien·ne, membre de l’équipe de recherche, de procéder à une revue de la médication du·de la participant·e âgé·e en collaboration avec le médecin désigné.

La **troisième étape (semaine 2)** consiste en une consultation menée par l’infirmier·ère membre du projet avec la personne âgée de 65 ans et plus et son·sa proche aidant·e. Cet entretien d’approximativement 45 minutes explorera leurs besoins et objectifs de soin, dans le but de réduire le risque de problèmes médicamenteux. À la suite de cette rencontre, un plan d'enseignement ciblé est conçu en collaboration avec le professionnel de la santé désigné. L’objectif de ce plan est de promouvoir l’engagement dans la réduction des risques de problèmes médicamenteux.

La **quatrième étape** **(semaine 3 et 4)** consiste à implémenter ce plan avec la personne âgée de 65 ans et plus et son·sa proche aidant·e au travers de deux consultations d’approximativement 45 minutes chacune à nouveau menées par l’infirmier·ère membre du projet.

La **cinquième étape (semaine 5)** consiste à faire un « état des lieux » avec la personne âgée de 65 ans et plus et son·sa proche aidant·e du chemin accompli. L’infirmier·ère membre du projet vous transmettra un questionnaire sur votre appréciation en termes d’acceptation et faisabilité du processus.

# **Bénéfices**

Votre participation au projet ne vous apportera aucun bénéfice. Toutefois, en participant, vous favoriserez à une meilleure compréhension des enjeux liés à la gestion médicamenteuse à domicile. Vous contribuerez à l’élaboration d’un programme interprofessionnel visant à réduire le risque d’évènements indésirables médicamenteux, en promouvant l’engagement actif des personnes âgées et proches aidants dans la gestion du traitement médicamenteux.

# **Caractère facultatif de la participation et obligations**

Votre participation est entièrement libre. Si vous choisissez de ne pas participer ou si vous choisissez de participer et revenez sur votre décision pendant le déroulement du projet, vous n’aurez pas à vous justifier. Cette décision n’aura pas de répercussions défavorables sur la suite de votre prise en charge médicale.

Si vous choisissez de participer à ce projet de recherche, vous serez tenu·e à rencontrer une fois durant approximativement 45 minutes un membre de l’équipe de recherche et, si possible pour vous, de contribuer au développement d’un plan d’enseignement pour réduire le risque de problèmes médicamenteux.

# **Risques et contraintes**

Cette étude ne comporte aucun risque pour vous. Néanmoins, les chercheurs ne peuvent pas exclure la possibilité que la personne âgée de 65 ans et pus ressente des altérations de son état habituel, si son médecin traitant décide d’adapter son traitement médicamenteux pour éviter des problèmes liées à la médication, à la suite de la discussion avec le pharmacien partenaire de l'étude.

# **Alternatives**

Si vous ne souhaitez pas participer à ce projet de recherche, mais vous restez ouvert à la possibilité d’une participation à d’autres projets, merci de l’indiquer l’infirmier·ère membre du projet.

# **Résultats**

Le projet permet d’obtenir différents résultats :

1. des résultats individuels concernant la personne âgée de 65 ans et plus,
2. des résultats définitifs objectifs du projet dans son ensemble.

1. L’infirmier·ère membre du projet avisera la personne âgée de 65 ans et plus pendant le projet de toute nouvelle découverte importante la concernant. Elle sera informée oralement et par écrit et pourra par la suite à nouveau décider s’elle souhaite poursuivre sa participation au projet.

2. L’infirmier·ère membre du projet peut vous faire parvenir, à l’issue du projet, une synthèse des résultats globaux.

# **Confidentialité des données et des échantillons**

- 1. **Traitement et codage des données**

Dans le cadre de ce projet de recherche, des données relatives à votre personne sont recueillies et traitées, en partie de manière automatisée. Ces informations sont codées au moment du relevé. Le codage signifie que toutes les données permettant de vous identifier (nom, date de naissance, etc.) sont remplacées par un code. Il n’est pas possible de relier les données à votre personne sans le code, qui reste en permanence au sein la Haute École de Santé, de la HES-SO Valais/ Wallis.

Seul un nombre limité de personnes peut consulter vos données sous une forme non codée, et ce, exclusivement afin de pouvoir accomplir des tâches nécessaires au déroulement du projet. Ces personnes sont tenues au secret professionnel. En tant que participant·e, vous avez à tout moment le droit de consulter vos données.

- 1. **Protection des données et des échantillons**

Toutes les directives relatives à la protection des données sont rigoureusement respectées. Il est possible que vos données doivent être transmises sous forme codée, par exemple pour une publication, et qu'elles puissent être mises à la disposition d'autres chercheur.e.s.

- 1. **Protection des données en cas de réutilisation**

Vos données pourraient ultérieurement se révéler importants pour répondre à d’autres questionnements et/ou être envoyées à une autre une banque de données située en Suisse ou à l’étranger pour être aussi exploités dans d’autres projets de recherche. Cette banque de données doit toutefois obéir aux mêmes normes et exigences que la banque de données du présent projet. Pour cette réutilisation, nous vous prions de signer une déclaration de consentement séparée à la fin de cette feuille d’information. Ce deuxième consentement est indépendant de la participation au projet.

- 1. **Droit de consultation dans le cadre d’inspections**

Le projet peut faire l’objet d’inspections. Celles-ci peuvent être effectuées par la commission d’éthique compétente. L’investigateur principal doit alors communiquer vos données pour les besoins de ces inspections. Toutes les personnes impliquées sont tenues au plus strict secret professionnel.

# **Retrait du projet**

Vous pouvez à tout moment vous retirer du projet si vous le souhaitez. Cependant, les données recueillis jusque-là pourront encore être analysés sous forme codée.

Après l’analyse, nous rendrons vos données anonymes. Nous effacerons définitivement le code les reliant à vous, si bien que plus personne ne pourra savoir après cela que ces données sont les vôtres. Ce procédé vise avant tout à assurer la protection de données.

# **Rémunération**

Vous ne percevrez aucune rémunération pour votre participation à ce projet de recherche.

Votre participation n’aura aucune conséquence financière pour vous ou votre assurance maladie.

# **Responsabilité**

La Haute École de Santé, de la HES-SO Valais/ Wallis qui a initié le projet de recherche et est chargée de sa réalisation est responsable des dommages que vous pourriez subir en relation avec le projet. Les conditions et la procédure sont fixées par la loi.

# **Financement**

Le projet est intégralement financé par la Haute École de Santé, de la HES-SO Valais/ Wallis.

# **Interlocuteur(s)**

Vous pouvez à tout moment poser des questions au sujet du projet. En cas d’incertitudes pendant ou après le projet, vous pouvez vous adresser à :

**Responsable du projet :**

Prof. Henk Verloo

Haute École de Santé, HES-SO Valais/ Wallis

Chemin de l’Agasse 5, 1950 Sion

Email : [henk.verloo@hevs.ch](mailto:henk.verloo@hevs.ch)

Tel. +41 58 606 84 24

**Collaborateurs :**

Mme Sofia Fernandes, chargée de cours

Haute École de Santé, HES-SO Valais/ Wallis

Chemin de l’Agasse 5, 1950 Sion

Email: [sofia.fernandes@hevs.ch](mailto:sofia.fernandes@hevs.ch)

Tel. +41 58 606 86 17

Prof. HES Ass. Filipa Pereira

Haute École de Santé, HES-SO Valais/ Wallis

Chemin de l’Agasse 5, 1950 Sion

Email : [filipa.pereira@hevs.ch](mailto:filipa.pereira@hevs.ch)

Tel. +41 58 606 84 42

Dr. Carla Meyer-Massetti, Pharmacienne

Institute for Primary Health Care BIHAM, Université de Bern

Mittelstrasse 43, CH-3012 Bern, Switzerland

Email: [carla.meyer-massetti@biham.unibe.ch](mailto:carla.meyer-massetti@biham.unibe.ch)

**Déclaration de consentement**

**Déclaration de consentement écrite pour la participation à un projet de recherche**

Veuillez lire attentivement ce formulaire. N’hésitez pas à poser des questions lorsque vous ne comprenez pas quelque chose ou que vous souhaitez avoir des précisions. Votre consentement écrit est nécessaire pour participer au projet.

| **Numéro BASEC du projet de recherche:** | 2021-01843 |
| --- | --- |
| **Titre (scientifique et usuel) :** | Optimisation de la gestion médicamenteuse pour des personnes âgées de 65 ans et plus polymédiquées vivant à domicile : l’étude pilote OptiMed |
| **Institution responsable (responsable du projet et adresse complète) :** | Haute École de Santé, HES-SO Valais/ Wallis  Chemin de l’Agasse 5, 1950 Sion  Email : henk.verloo@hevs.ch  Tel. +41 58 606 84 24 |
| **Lieu de réalisation :** | Canton du Valais |
| **Responsable du projet sur le site :** Nom et prénom en caractères d’imprimerie : | Prof. Henk Verloo |
| **Participante / Participant :** Nom et prénom en caractères d’imprimerie : Date de naissance : |  |

- Je déclare avoir été informé·e, par l’investigateur/investigatrice soussigné·e, oralement et par écrit, des objectifs et du déroulement du projet de recherche ainsi que des avantages et des inconvénients possibles et des risques éventuels.
- Je prends part à ce projet de façon volontaire et j’accepte le contenu de la feuille d’information qui m’a été remise sur le projet précité. J’ai eu suffisamment de temps pour prendre ma décision.
- J’ai reçu les réponses aux questions que j’ai posées en relation avec la participation à ce projet. Je conserve la feuille d’information et reçois une copie de ma déclaration de consentement écrite.
- J’accepte que les spécialistes compétent·e·s de la direction de ce projet et de la commission d’éthique compétente puissent consulter mes données non codées afin de procéder à des contrôles et des inspections, à condition toutefois que la confidentialité de ces données soit strictement assurée.
- Je sais que mes données personnelles peuvent être transmis(es) à des fins de recherche dans le cadre de ce projet et uniquement sous une forme codée. Le promoteur assure une protection des données conforme aux normes et exigences suisses.
- Je peux, à tout moment et sans avoir à me justifier, révoquer mon consentement à participer au projet, sans que cette décision n’ait de répercussions défavorables sur la suite de ma prise en charge. Les données recueillies jusqu’au retrait seront cependant analysées dans le cadre du projet.
- Je suis informé·e que la Haute École de Santé, HES-SO Valais/ Wallis est responsable des dommages éventuels imputables au projet.

| Lieu, date | Signature de la participante / du participant |
| --- | --- |

**Attestation de l’investigateur/investigatrice :** Par la présente, j’atteste avoir expliqué à la participante / au participant la nature, l’importance et la portée du projet. Je déclare satisfaire à toutes les obligations en relation avec ce projet conformément au droit suisse en vigueur. Si je devais prendre connaissance, à quelque moment que ce soit durant la réalisation du projet, d’éléments susceptibles d’influer sur le consentement de la participante / du participant à prendre part au projet, je m’engage à l’en informer immédiatement.

| Lieu, date | Nom et prénom l’investigateur/investigatrice en caractères d’imprimerie  Signature de l’investigateur/investigatrice |
| --- | --- |

**Déclaration de consentement écrite pour la réutilisation de données sous une forme codée**

| **Numéro BASEC du projet de recherche :** | 2021-01843 |
| --- | --- |
| **Titre (scientifique et usuel) :** | Optimisation de la gestion médicamenteuse pour des personnes âgées de 65 ans et plus polymédiquées vivant à domicile : l’étude pilote OptiMed |
| **Participante / Participant :** Nom et prénom en caractères d’imprimerie : Date de naissance : |  |

J’accepte que mes données obtenues dans le cadre de ce projet puissent être réutilisés à des fins de recherche médicale sous forme codée. Cela signifie que mes données seront conservées et ultérieurement exploitées pour une durée indéfinie, tant que je ne retire pas mon consentement, dans le cadre de futurs projets de recherche.

Je sais que mes données sont conservées sous forme codée et que la liste d’identification est gardée dans un lieu sûr. Les données peuvent être envoyées à des fins d’analyse à une autre banque de données située en Suisse ou à l’étranger, à condition qu’elle obéisse à des normes et exigences au moins équivalentes aux normes et exigences suisses. Toutes les dispositions légales relatives à la protection des données sont respectées.

Je donne mon accord de façon volontaire et je peux à tout moment revenir sur ma décision. Si je reviens sur ma décision, mes données seront rendues anonymes. Je dois simplement en informer la personne responsable du projet. Je n’ai pas à justifier ma décision. Dans ce cas, j’accepte que mes données soient anonymisées et j’ai compris que je ne pourrai pas être informé des résultats ni retirer mon consentement ultérieurement.

Généralement, les données sont exploitées de manière globale et les résultats sont publiés de manière synthétique. Il se peut que je sois contacté·e si l’analyse des données révèle une découverte pertinente pour ma santé. Si je ne souhaite pas en être informé·e, il m’incombe d’en aviser la personne responsable du projet.

| Lieu, date | Signature de la participante / du participant |
| --- | --- |

**Attestation de la personne investigatrice :** Par la présente, j’atteste avoir expliqué à la participante / au participant la nature, l’importance et la portée de la réutilisation de données.

| Lieu, date | Nom et prénom de la personne investigatrice en caractères d’imprimerie.  Signature de la personne investigatrice |
| --- | --- |
