# Supplementary material for: An Intervention Program to Reduce Medication-Related Problems Among Polymedicated Home-Dwelling Older Adults (OptiMed): Protocol for a Pre-Post, Multisite, Pilot, and Feasibility Study
Source: JMIR Res Protoc. 2023 Jan 25;12:e39130. doi: 10.2196/39130 (PMC9909524; doi:10.2196/39130)
Supplement: Multimedia Appendix 4 [file resprot_v12i1e39130_app4.docx]

Supplementary File 4*.* List of potential outcomes to include in Optimed’s full-scale study after the pilot study, based on the Core Outcome Set reported by Beuscart et al. (49).

| **Domain** | **Outcome** | **Definition** |
| --- | --- | --- |
| Medication reconciliation^1^ | Number of discrepancies | Number of discrepancies between professional sources; number of discrepancies between patient-reported medication use and professional sources. |
|  | Number of clarifications needed | Number and type of clarifications needed due to missing prescription information. |
| Medication analysis^1^ | Overuse | The use or prescription of more medications than clinically needed, including: 1) any medication prescribed or used without an evidence-based clinical indication; 2) therapeutic duplication; 3) medication prescribed or used beyond the recommended duration. |
|  | Underuse | A failure to prescribe medications that are indicated, including: 1) omission of an evidence-based medication; 2) too short a duration; 3) dosage too low based on international recommendations and evidence-based guidelines. |
|  | Potentially inappropriate medications | Medications with a risk of adverse drug reactions exceeding their expected clinical benefit to patients, particularly when safer therapeutic alternatives are available to treat the same condition. |
|  | Clinically significant drug–drug interactions | Significant severity rating according to the medication interaction compendia used in the study. |
| Adverse events^1^ | Medication-related hospital admissions | Hospitalisation due to an adverse medication event: harm due to an adverse medication reaction or a medical error related to overuse, underuse or misuse of prescription and non-prescription medications, and which is the main reason for, or contributes to, a patient’s hospital admission. |
|  | Medication-related problems (MRPs) | Numbers and types of MRPs, such as adverse medication reactions, medication errors and hospital (re)admissions (4, 64). |
| Patient-reported outcomes^2^ | Able to stay at home?  Quality of life? | PROS, PROMS and PREMS (47) will be defined by the pilot study. |

^1^ Primary outcomes. ^2^ Secondary outcomes.
