# Supplementary material for: An Intervention Program to Reduce Medication-Related Problems Among Polymedicated Home-Dwelling Older Adults (OptiMed): Protocol for a Pre-Post, Multisite, Pilot, and Feasibility Study
Source: JMIR Res Protoc. 2023 Jan 25;12:e39130. doi: 10.2196/39130 (PMC9909524; doi:10.2196/39130)
Supplement: Multimedia Appendix 5 [file resprot_v12i1e39130_app5.docx]

**Questionnaire de récolte de données pour les participants âgés**

Code du participant :_____________________

Date de la récolte de données :_____________________

**Données sociodémographiques et de santé**

1. Année de naissance: ____
2. Genre : □ Masculin □ Féminin □ Autre
3. Avec qui partagez-vous votre logement :

□ Seul·e □ Conjoint·e □ Enfant(s) □ Autre: _________________

1. Nombre d’hospitalisations et d’admissions aux urgences au cours des 12 derniers mois: _________
2. Motifs : ___________________________________________________________________
3. Date de la dernière admission à l’hôpital ou aux urgences ____/ ____/ _____
4. Date du dernier retour à domicile ___ /____/____
5. Nombre de consultations médicales au cours des 12 derniers mois : _________
6. Quel professionnel de la santé a été consulté ?

□ Médecin généraliste □ Cardiologue □ Diabétologue

□ Gériatre □ Psychiatre

□ Infirmier·ère ou infirmier·ère spécialisé·e

□ Autre : _______________________________________________

1. Qui vous a prescrit des médicaments ?

□ Médecin généraliste □ Cardiologue □ Diabétologue

□ Gériatre □ Psychiatre

□ Autre : _______________________________________________

1. Avez-vous une pharmacie ou un pharmacien personnel ?

□ Non □ Oui, un.

□ Oui, plusieurs (combien ?) : _____________

1. Comorbidités (diagnostics ICD-10):

*[À compléter par le centre de soins à domicile et le participant âgé]* ______________________________________________________________________________________________________________________________________________________________________________________________________________________________________________________

**Gestion de la médication**

1. Liste des médicaments déclarés par la personne âgée :

*[Si possible, le chercheur devrait compléter ce rapport avec une copie ou une photographie d’une liste à jour des médicaments de la personne âgée, ou solliciter l’aide du centre de soins à domicile]*

______________________________________________________________________________________________________________________________________________________________________________________________________________________________________________________

1. Qui est responsable de la préparation de vos médicaments ?

□ Moi

□ Mon proche aidant. Lien de parenté : _________________

□ Mon soignant formel : ________________________________________

1. Actuellement, prenez-vous un ou plusieurs médicaments non-prescrits ?

□ Non □ Oui

1. Actuellement, combien de médicaments non-prescrits prenez-vous **par jour**? ____________
2. Qu’est-ce qui pourrait vous aider à gérer votre médication de manière plus efficace (afin d’optimiser votre gestion médicamenteuse, par exemple un entraînement, une aide à la distribution des médicaments, des applications sur le téléphone, etc.) ?

______________________________________________________________________________________________________________________________________________________________________________________________________________________________________________________

**Six-item Cognitive Impairment Test (6-CIT)**

1. En quelle année sommes-nous ?

0

4

Correct Incorrect

1. Quel mois somme-nous ?

3

0

Correct Incorrect

Souvenez-vous de l’adresse suivante : Pierre/Martin/42/Rue des Accacias/Neuilly

1. Quelle heure est-il ?

3

0

Correct Incorrect

1. Comptez à rebours de 20 à 1.

4

2

0

Correct Incorrect Plus de 1 erreur

1. Dites les mois de l’année à l’envers.

0

2

4

Correct Incorrect Plus de 1 erreur

1. Répétez l’adresse.

4

0

2

Correct 1 erreur 2 erreurs

100

8

6

3 erreurs 4 erreurs 5 erreurs

Résultats :

0-7 normal / 8-9 présence de troubles cognitifs légers / 10 -28 présence de troubles cognitifs significatifs.

**Tilburg Frailty Indicator (TFI)**

Gobbens RJJ, Schols JMGA, Van Assen MALM. Exploring the efficiency of the Tilburg Frailty indicator: A review. Clinical Interventions in Aging. 2017;12:1739-52.

| **Section A : Déterminants de la fragilité** |
| --- |
| 1. Quel est votre sexe ?   🔾 Homme  🔾 Femme |
| 1. Quel est votre âge ? ……….années |
| 1. Quel est votre état civil ?   🔾 Marié ou concubinage  🔾 Célibataire  🔾 Divorcé  🔾 Veuf / veuve |
| 1. Quel est votre lieu de naissance ?   🔾 Suisse  🔾 Portugal  🔾 Espagne  Autre :……….. |
| 1. Quel est votre niveau d’éducation le plus élevé que vous avez suivi ?   🔾 Pas de formation ou école primaire (0 à 6 ans)  🔾 École secondaire (7 à 12 ans)  🔾 Apprentissage  🔾 Haute école spécialisée ou université |
| 1. Quel est votre revenu mensuel pour le ménage ?   🔾 Inférieur à 1’500 CHF  🔾 CHF 1’500 à 2’500 CHF  🔾 Supérieur à 2’500 CHF |
| 1. Comment évaluez-vous votre niveau de santé ?   🔾 En bonne santé  🔾 Ni en bonne santé et ni en mauvaise santé  🔾 En mauvaise santé |
| 1. Avez-vous deux ou plusieurs maladies chroniques ?   🔾 Oui  🔾 Non |
| 1. Avez-vous la dernière année vécue un ou plusieurs de ces événements ?  - Décès d’une personne proche/ami ? - Une maladie vous affectant personnellement ? - Une maladie affectant l’un de vos proches/amis ? - Un divorce, fin d’une relation intime de longue durée ? - Un accident de la circulation ? - Un crime/délit ?   🔾 Oui  🔾 Non |
| 1. Êtes-vous satisfait de votre habitat ?   🔾 Oui  🔾 Non |
| **Partie B : Déterminants de fragilité**  **Composantes physiques B1** |
| 1. Vous sentez-vous physiquement en bonne santé ?   🔾 Oui  🔾 Non |
| 1. Avez-vous perdu du poids durant ces 6 derniers mois sans le vouloir ?   (Beaucoup = 6kg ou plus au cours des 6 derniers mois / 3kg ou plus au cours du mois passé)  🔾 Oui  🔾 Non |
| Éprouvez-vous des difficultés dans votre vie quotidienne :   1. Des difficultés à marcher ? 2. Des difficultés à garder votre équilibre ? 3. Des troubles de l’audition ? 4. Des troubles de la vue ? 5. Des faiblesses dans les mains ? 6. De l’épuisement physique ?   🔾 Oui  🔾 Non |
| **B2 Composantes psychiques** |
| 1. Avez-vous des plaintes concernant votre mémoire ?   🔾 Oui  🔾 Parfois  🔾 Non |
| 1. Avez-vous eu ce dernier mois des troubles de l’humeur ?   🔾 Oui  🔾 Parfois  🔾 Non |
| 1. Avez-vous eu ce dernier mois des moments de nervosité ou d’anxiété ?   🔾 Oui  🔾 Parfois  🔾 Non |
| 1. Êtes-vous capable de gérer vos problèmes ?   🔾 Oui  🔾 Non |
| **B3 Composantes sociales** |
| 1. Vivez-vous seul ?   🔾 Oui  🔾 Non |
| 1. Manquez-vous de personnes qui vous entourent ?   🔾 Oui  🔾 Parfois  🔾 Non |
| 1. Recevez-vous assez de soutien d’autres personnes ?   🔾 Oui  🔾 Non |
| **Seuil du score fragilité : ≥ 5 points**  Questions 11, 22, 25: Oui= 0; Non = 1  Questions 12 jusqu’au 18, 23 : Non = 0, Oui = 1  Question 19: non / parfois = 0, Oui= 1  Questions 20, 21, 24: Non=0, Oui/parfois: 1 |

**doMESTIC RISK**

| **Nr.** | **Facteur de risque** | **Score** | **Résultat** |
| --- | --- | --- | --- |
| **1** | Patient(e) confiné à son domicile avec des problèmes cognitifs et en manque de soutien social | **1 point** |  |
| **2** | Plusieurs prescripteurs en même temps ou prescripteurs changeants  (hôpital, établissement réhabilitation, médecin traitant, spécialiste) | **2 points** |  |
| **3** | Transfert récent de l’hôpital vers les soins à domicile | **1 point** |  |
| **4** | Dysfonctionnement rénal DFG < 30ml/min | **2 points** |  |
| **5** | Nombre de médicaments ≥ 7 (y compris les réserves) | **2 points** |  |
| **6** | Médicaments à haut risque/groupe de médicaments et/ou médicaments à marge thérapeutique étroite  (Neuroleptiques, notamment Lithium, Digoxine, Amiodaron et autres antiarythmiques, antiépileptiques, notamment Phenytoin, Phenobarbital, Carbamazepin, anticoagulants oraux, notamment Marcoumar, ACODs (anticoagulants oraux directs), Insuline, Méthotrexate, Theophylline)  Et/ou  ≥ 3 médicaments avec effet sur le système nerveux central (médicaments à action centrale : analgésique, antipsychotique, antidépresseurs, Benzodiazépine) | **1 point** |  |
| **7** | Médicaments ne convenant pas aux patients gériatriques  (p. ex. liste Priscus®) | **3 point** |  |
| **8** | Double prescription médicale d’une substance (p. ex générique et original) ou d’une classe de médicaments | **3 points** |  |
| **9** | Patient – manque de compréhension de la thérapie et/ou de la maladie (problèmes de cognition ou/et de communication, p. ex. langue étrangère, déficience auditive) | **1 point** |  |
| **10** | Non-Adhésion / manque de conformité/compliance/observance thérapeutique (p. ex. absorption incorrecte par manque de compréhension de la thérapie)  Et/ou  Le patient prend des médicaments sans que le médecin soit au courant (également médicaments achetés lui-même) | **1 point** |  |

Résultat final de l’instrument:

Score plus petit que 5 : en principe pas besoin d’intervenir
contrôle de la médication par la pharmacienne des soins à domicile sur demande de l’infirmière en charge du patient

Score 5 ou plus : vive recommandation d’analyser la médication par le pharmacien des soins à domicile

**Acceptabilité**

| **Items** | **Niveau d’acceptabilité**  **0 = pas du tout acceptable**  **10 = complètement acceptable** |
| --- | --- |
| **Évaluation initiale :**  Questionnaire de récolte de données  6-CIT  TFI  ICD-10  Liste de médicaments  Évènements indésirables médicamenteux préalables  doMESTIC RISK | 0 – 1 – 2 – 3 – 4 – 5 – 6 – 7 – 8 – 9 – 10  0 – 1 – 2 – 3 – 4 – 5 – 6 – 7 – 8 – 9 – 10  0 – 1 – 2 – 3 – 4 – 5 – 6 – 7 – 8 – 9 – 10  0 – 1 – 2 – 3 – 4 – 5 – 6 – 7 – 8 – 9 – 10  0 – 1 – 2 – 3 – 4 – 5 – 6 – 7 – 8 – 9 – 10  0 – 1 – 2 – 3 – 4 – 5 – 6 – 7 – 8 – 9 – 10  0 – 1 – 2 – 3 – 4 – 5 – 6 – 7 – 8 – 9 – 10 |
| **Évaluation finale :**  doMESTIC RISK  Hospitalisation/ admissions aux urgences | 0 – 1 – 2 – 3 – 4 – 5 – 6 – 7 – 8 – 9 – 10  0 – 1 – 2 – 3 – 4 – 5 – 6 – 7 – 8 – 9 – 10 |
| **Intervention t1** | 0 – 1 – 2 – 3 – 4 – 5 – 6 – 7 – 8 – 9 – 10 |
| **Intervention t2** | 0 – 1 – 2 – 3 – 4 – 5 – 6 – 7 – 8 – 9 – 10 |
| **Intervention t3** | 0 – 1 – 2 – 3 – 4 – 5 – 6 – 7 – 8 – 9 – 10 |
