# Supplementary material for: An Intervention Program to Reduce Medication-Related Problems Among Polymedicated Home-Dwelling Older Adults (OptiMed): Protocol for a Pre-Post, Multisite, Pilot, and Feasibility Study
Source: JMIR Res Protoc. 2023 Jan 25;12:e39130. doi: 10.2196/39130 (PMC9909524; doi:10.2196/39130)
Supplement: Multimedia Appendix 6 [file resprot_v12i1e39130_app6.docx]

**Questionnaire de récolte de données pour les proches aidants**

Code du participant : _________

Date de la récolte de données : _______________

1. Année de naissance : _______
2. Genre : □ Féminin □ Masculin □ Autre
3. Niveau de formation : □ École obligatoire □ École secondaire

□ Études supérieures

1. Statut professionnel : □ Employé·e □ Retraité·e

□ Incapacité de travailler/handicap □ Sans emploi

1. Distance du proche aidé : □ Logement partagé □ < 20 min □ > 20 min
2. Moyen de transport utilisé pour se rendre chez le proche aidé :

□ Voiture privée □ À pied □ Transports publiques

□ Autre :_____________

1. Comment contribuez-vous à la gestion de la médication de votre proche ?

| Activités liées à la gestion de la médication | | Fréquence de la participation et de l’activité | | | Qualité de l’information | | | |
| --- | --- | --- | --- | --- | --- | --- | --- | --- |
|  |  | Rarement/jamais | Parfois | La plupart des jours / chaque jour | Aucune | Insuffisante | Bonne | Très bonne |
| Activités directement liées à la gestion de la médication | Demander des ordonnances au médecin |  |  |  |  |  |  |  |
|  | Commander la médication |  |  |  |  |  |  |  |
|  | Récupérer la médication |  |  |  |  |  |  |  |
|  | Diviser ou couper des pilules ou préparer la médication sous forme liquide |  |  |  |  |  |  |  |
|  | Préparer des piluliers ou des aides à la distribution de la médication |  |  |  |  |  |  |  |
|  | Aider à l’administration de la médication |  |  |  |  |  |  |  |
|  | S’assurer que la médication est prise comme prescrit |  |  |  |  |  |  |  |
| Activités indirectement liées à la gestion de la médication | Surveiller les événements indésirables ou les effets secondaires |  |  |  |  |  |  |  |
|  | Stocker la médication |  |  |  |  |  |  |  |
|  | Recueillir des informations sur la médication |  |  |  |  |  |  |  |
|  | Interagir avec des membres du système de soin |  |  |  |  |  |  |  |
|  | Prendre des décisions quant au traitement |  |  |  |  |  |  |  |
|  | Accompagner aux consultations de santé |  |  |  |  |  |  |  |
|  | Clarifier les questions avec les professionnels de la santé (par téléphone, e-mail, etc.) |  |  |  |  |  |  |  |
| Outils | Gérer les piluliers, les coupe-pilules et les dispositifs permettant de mesurer correctement la médication sous forme liquide |  |  |  |  |  |  |  |
|  | Préparer et gérer la liste de médication |  |  |  |  |  |  |  |
|  | Gérer les listes de rappel, les calendriers et autres rappels |  |  |  |  |  |  |  |
| Stratégies | Rappeler à la personne âgée |  |  |  |  |  |  |  |
|  | Intégrer la médication dans la routine quotidienne |  |  |  |  |  |  |  |
|  | Utiliser plusieurs récipients et bouteilles |  |  |  |  |  |  |  |
|  | Réduire l’accès de la personne âgée à la médication |  |  |  |  |  |  |  |
|  | Étiqueter le pilulier |  |  |  |  |  |  |  |

•

1. Qu'est-ce qui pourrait vous aider à optimiser la gestion médicamenteuse de votre proche (en ce qui concerne vos tâches ou votre collaboration avec les professionnels de la santé) ?

____________________________________________________________________________________________________________________________________________________________________________________________________________________________________________________________________________________________

**Acceptabilité**

| **Items** | **Niveau d’acceptabilité**  **0 = pas du tout acceptable**  **10 = complètement acceptable** |
| --- | --- |
| Questionnaire de récolte de données pour les proches aidants  Rôle dans la gestion de la médication | 0 – 1 – 2 – 3 – 4 – 5 – 6 – 7 – 8 – 9 – 10  0 – 1 – 2 – 3 – 4 – 5 – 6 – 7 – 8 – 9 – 10 |
| **Intervention t1** | 0 – 1 – 2 – 3 – 4 – 5 – 6 – 7 – 8 – 9 – 10 |
| **Intervention t2** | 0 – 1 – 2 – 3 – 4 – 5 – 6 – 7 – 8 – 9 – 10 |
| **Intervention t3** | 0 – 1 – 2 – 3 – 4 – 5 – 6 – 7 – 8 – 9 – 10 |
