# Supplementary material for: An Intervention Program to Reduce Medication-Related Problems Among Polymedicated Home-Dwelling Older Adults (OptiMed): Protocol for a Pre-Post, Multisite, Pilot, and Feasibility Study
Source: JMIR Res Protoc. 2023 Jan 25;12:e39130. doi: 10.2196/39130 (PMC9909524; doi:10.2196/39130)
Supplement: Multimedia Appendix 7 [file resprot_v12i1e39130_app7.docx]

**Questionnaire de récolte de données pour les professionnels de la santé**

*[à compléter avant l’intervention, en se concentrant sur le patient âgé et non la population]*

Code du participant : ………………………

Date de la récolte de données : ……/………/….…

**Données sociodémographiques et professionnelles**

1. Année de naissance :_______
2. Genre : □ Féminin □ Masculin □ Autre
3. Profession : □ Infirmier·ère □ Médecin □ Pharmacien·ne
4. Lieu de travail : ____________________________________
5. Taux de travail (nombre d’heures de travail par semaine) : _________________
6. Ancienneté dans la profession (expérience professionnelle dans ce champ) : ____ (année)
7. Formation en soins gériatriques ?

□ Non □ Oui (merci de décrire) : ____________________________

1. Formation en pharmacologie gériatrique et/ou en gestion médicamenteuse à domicile ?

□ Non □ Oui (merci de décrire) : ____________________________

**Contrôle et suivi de la médication**

1. Votre lieu de travail fournit-il un formulaire de suivi de la prise de médication pour les patients âgés ? □ Non □ Oui
2. Si oui, quels outils de suivi de la prise de médication avez-vous utilisé/utilisez-vous avec les patients âgés ? □ Formulaire □ Appels téléphoniques

□ Autre: ____________

1. Si oui, avez-vous l’habitude de consulter son historique pour confirmer le traitement à administrer ?  □ Non □ Oui

**Enseignement et formation fournis sur la gestion de la médication**

1. Avez-vous enseigné ou formé les patients âgés et/ou leurs proches aidants à la gestion des médicaments avant le début de l'étude ? □ Non □ Oui
2. Quels aspects avez-vous l’habitude d’inclure dans cet enseignement ou cette formation avant le début de l’étude ?

|  | **Chaque consultation ou visite à domicile** | **Lorsqu’un nouveau médicament était prescrit** | **Lorsque la médication en cours a fait l’objet d’un ajustement de dosage** | **Lorsque la personne le demandait** | **Jamais** |
| --- | --- | --- | --- | --- | --- |
| 1. L’objectif de chaque médicament |  |  |  |  |  |
| 1. Dosage et fréquence |  |  |  |  |  |
| 1. Exigences en lien avec l’administration (à jeun vs. pré ou post-prandial), technique (ex. insuline) |  |  |  |  |  |
| 1. Effets indésirables |  |  |  |  |  |
| 1. Interactions (ex. avec la médication prescrite et non-prescrite, nourriture) |  |  |  |  |  |
| 1. Stockage |  |  |  |  |  |
| 1. Stratégies pour améliorer la gestion médicamenteuse (ex. pilulier, calendrier, autre) |  |  |  |  |  |
| 1. Facteurs favorisant l’adhésion (ex. motivation, rappels) |  |  |  |  |  |
| 1. Capacité économique à se procurer le médicament |  |  |  |  |  |
| 1. Autre (merci de décrire)   ___________________________ |  |  |  |  |  |

1. Fournissez-vous aux patients âgés et/ou à leurs soignants informels une **liste écrite ou imprimée de la médication à chaque changement de médicament** ?

□ No □ Yes

1. Si oui, quelles informations figurent sur cette liste?

| □ Le nom du patient  □ Les médicaments modifiés  □ Le traitement entier  □ Le dosage  □ Planning de la prise de traitement | □ L’indication thérapeutique  □ Les mesures de sécurité  □Coordonnées de l’infirmier·ère traitant·e (ex. numéro de téléphone)  □ Coordonnées du/de la pharmacien·e  □ Coordonnées du médecin généraliste ou du/de la spécialiste |
| --- | --- |

**Evaluation des capacités cognitives pour la gestion de la médication**

1. Lorsque la médication **est** modifiée, **évaluez-vous si la personne âgée a la capacité cognitive** de comprendre et de gérer ce médicament en association avec d'autres médicaments en cours ?

□ Non □ Oui

1. Si oui, procédez-vous habituellement à cette évaluation en utilisant une **échelle validée de littératie en matière de santé ou de fonctions cognitives** ?

□ Non □ Oui (merci de décrire): _____________________

1. Si la personne âgée **n'a pas la capacité cognitive** de comprendre et de gérer sa nouvelle ordonnance ou son association avec d'autres médicaments en cours, que faites-vous ?

□ Changer la prescription (médecin généraliste) ou suggérer au prescripteur de changer la prescription

□ Ajuster les autres médicaments

□ Demander à un soignant informel d’aider à gérer la nouvelle prescription

□ Demander de l’aide à d’autres professionnels de la santé (qui ?) : _____________

______________________________________________________________________

□ Autre : ____________________________________________________________

**Collaboration avec les intervenants**

1. Quand vous doutez de l’adéquation d’une prescription (dosages, horaires de traitement, interactions possibles), à quelle fréquence contactez-vous les autres prescripteurs (médecin généraliste, hôpital, médecin spécialiste) ?

□ Toujours □ Parfois □ Jamais □ Cela dépend du prescripteur

1. La communication est-elle facile ?

□ Oui □ Cela dépend du prescripteur □ Non (merci de décrire pourquoi): ____________________________________________________________

1. Quand vous consultez une personne âgée après une urgence ou une consultation avec le spécialiste, mentionnez-vous l’importance d’informer le médecin généraliste (médecin de famille) concernant la nouvelle médication ? □ Non □ Oui
2. Transmettez-vous cette information ? □ Non □ Oui
3. Avez-vous des suggestions (issues de votre expérience clinique ou de votre pratique) qui pourraient améliorer la collaboration entre les intervenants pour la gestion médicamenteuse des personnes âgées à domicile ?

_________________________________________________________________________________________________________________________________________________________________________________________________________________________________

**Gestion de la médication**

1. Dans votre travail quotidien, avez-vous rencontré des **obstacles à la participation du patient âgé dans la gestion de sa médication** ? Merci de les décrire :

Au niveau des patients : ____________________________________________________

Au niveau des proches aidants : ___________________________________________

Au niveau interprofessionnel : _______________________________________________

Au niveau des procédures : __________________________________________________

Au niveau de l’utilisation des équipements/matériel disponible : ________________________________________________________________________

Autre : _____________________________________________________________

____________________________________________________________________

1. Souhaiteriez-vous recevoir **plus de formation sur la gestion de la médication pour les personnes âgées vivant à domicile** ? □ Non □ Oui
2. Qu'est-ce qui serait utile à cet égard pour votre pratique clinique ? _____________________________________________________________________________________________________________________________________________________________________________________________________________________
3. Avez-vous des suggestions (issues de votre expérience clinique ou de votre pratique) sur la manière de prévenir les problèmes liés à la médication chez les personnes âgées polymédicamentées vivant à domicile ?

____________________________________________________________________________________________________________________________________________________________________________________________________________________________________________________________________________________________________________

**Acceptabilité**

| **Items** | **Niveau d’acceptabilité**  **0 = pas du tout acceptable**  **10 = complètement acceptable** |
| --- | --- |
| Questionnaire de récolte de données | 0 – 1 – 2 – 3 – 4 – 5 – 6 – 7 – 8 – 9 – 10 |
| Intervention t1 (en cas de participation) | 0 – 1 – 2 – 3 – 4 – 5 – 6 – 7 – 8 – 9 – 10 |
| Intervention t2 (en cas de participation) | 0 – 1 – 2 – 3 – 4 – 5 – 6 – 7 – 8 – 9 – 10 |
| Intervention t3 (en cas de participation) | 0 – 1 – 2 – 3 – 4 – 5 – 6 – 7 – 8 – 9 – 10 |
