# Supplementary material for: An Intervention Program to Reduce Medication-Related Problems Among Polymedicated Home-Dwelling Older Adults (OptiMed): Protocol for a Pre-Post, Multisite, Pilot, and Feasibility Study
Source: JMIR Res Protoc. 2023 Jan 25;12:e39130. doi: 10.2196/39130 (PMC9909524; doi:10.2196/39130)
Supplement: Multimedia Appendix 8 [file resprot_v12i1e39130_app8.docx]

**Questionnaire feasibility – OptiMED pilot study**

| **Recruitment** | **Result** |
| --- | --- |
| Number of participants screened: |  |
| Number of participants enrolled: |  |
| Average time between invitation and enrolment of participants: |  |
| Time needed to enrol participants in the pilot study (information to consent): |  |
| Reasons for refusing to participate: | |
| 1.  2.  3.  4.  5.  ... | |
| Average time between invitation and enrolment of informal caregivers; Mean, median: | |
| Average time between invitation and enrolment of primary care nurses; Mean, median: | |
| Average time between invitation and enrolment of pharmacists; Mean, median: | |

**Time spent on:**

| **Baseline Assessment** | **Min–Max** | **Mean/median** | **Comment** |
| --- | --- | --- | --- |
| Sociodemographic data assessment  eCRF |  |  |  |
| Health status  6-CIT  TFI  ICD-10 |  |  |  |
| Risk of MRPs  Getting access to or compiling the medication list  Gathering previous MRPs  Completing the doMESTIC RISK tool |  |  |  |
| Informal caregiver:  Sociodemographic data  Role in medication management |  |  |  |
| Nurse primary care manager:  Sociodemographic and professional data |  |  |  |
| Pharmacist:  Sociodemographic and professional data |  |  |  |
| Physician:  Sociodemographic and professional data |  |  |  |
| **Final Assessment** | **Min–Max** | **Mean/median** | **Comment** |
| **Risk of MRPs:**  doMESTIC RISK tool  **Numbers and types of medication changes made:** |  |  |  |

**Time spent applying the intervention**

| **Medication-management intervention programme assessment** | **Min–Max** | **Mean/median** | **Comment** |
| --- | --- | --- | --- |
| **Intervention t1**   - Reviewing prescribed medication using STOPP/START criteria (pharmacist) - Recommendations/questions/requests for clarification sent to physicians |  |  |  |
| **Intervention t2**   - Exploring older adults and informal caregivers’ needs and care goals to reduce older adults’ risks of MRPs - Designing a target education plan to empower older adults and informal caregivers’ medication management and promoting their active engagement in reducing risks of MRPs |  |  |  |
| **Intervention t3**   - Implementing the target plan to empower older adults and informal caregivers’ medication management and promoting their active engagement in reducing risks of MRPs |  |  |  |

**Adherence to intervention**

| **Interventions** | **Number of participants**  **adhering** | **Number of participants**  **not adhering** | **Comments,**  **difficulties or barriers** |
| --- | --- | --- | --- |
| **Intervention t1**   - Reviewing prescribed medication using STOPP/START criteria (pharmacist) - Recommendations/questions/requests for clarification sent to the physician |  |  |  |
| **Intervention t2**   - Exploring older adult and informal caregivers’ needs and care goals to reduce older adults’ risks of MRPs - Designing a target education plan to empower older adults and informal caregivers’ medication management and promoting their active engagement in reducing risks of MRPs |  |  |  |
| **Intervention t3**   - Implementing the target plan to empower older adults and informal caregivers’ medication management and promoting their active engagement in reducing risks of MRPs |  |  |  |
| **Ratio of adherent to not adherent participants:** | | | |

**Medication review**

| **Medication review** | **Number/participant** | **Comments** |
| --- | --- | --- |
| Number of discrepancies between professional sources |  |  |
| Number of discrepancies between patient-reported medication use and professional sources |  |  |
| Number of items needing clarification |  |  |
| Number of recommendations made by the pharmacist |  |  |
| Number of pharmacist’s recommendations adopted by the physician |  |  |

**Ease of use**

| **Ease of use** | **No changes needed** | **Changes needed** |
| --- | --- | --- |
| doMESTIC RISK tool’s ease of use |  |  |
| Structured medication review template’s ease of use |  |  |
| Intervention t1 |  |  |
| Intervention t2 |  |  |
| Intervention t3 |  |  |
| ….. |  |  |
| ….. |  |  |

| **Pilot study participant retention** | Result |
| --- | --- |
| The number of drop-outs: |  |
| Reasons for drop-outs: | |
| 1.  2.  3.  4.  5.  ... | |

**General acceptability**

| **Items** | **Level of acceptability**  **0 = not at all acceptable**  **10 = completely acceptable** |
| --- | --- |
| Baseline assessments  Assessment sociodemographic data  6-CIT  TFI  ICD-10  Medication list  Previous MRPs  doMESTIC RISK tool  Informal caregiver:  Sociodemographic data  Role in medication management  Nurse primary-care manager:  Sociodemographic and professional data  Pharmacist:  Sociodemographic and professional data  Physician:  Sociodemographic and professional data | 0 – 1 – 2 – 3 – 4 – 5 – 6 – 7 – 8 – 9 – 10  0 – 1 – 2 – 3 – 4 – 5 – 6 – 7 – 8 – 9 – 10  0 – 1 – 2 – 3 – 4 – 5 – 6 – 7 – 8 – 9 – 10  0 – 1 – 2 – 3 – 4 – 5 – 6 – 7 – 8 – 9 – 10  0 – 1 – 2 – 3 – 4 – 5 – 6 – 7 – 8 – 9 – 10  0 – 1 – 2 – 3 – 4 – 5 – 6 – 7 – 8 – 9 – 10  0 – 1 – 2 – 3 – 4 – 5 – 6 – 7 – 8 – 9 – 10  0 – 1 – 2 – 3 – 4 – 5 – 6 – 7 – 8 – 9 – 10  0 – 1 – 2 – 3 – 4 – 5 – 6 – 7 – 8 – 9 – 10  0 – 1 – 2 – 3 – 4 – 5 – 6 – 7 – 8 – 9 – 10  0 – 1 – 2 – 3 – 4 – 5 – 6 – 7 – 8 – 9 – 10  0 – 1 – 2 – 3 – 4 – 5 – 6 – 7 – 8 – 9 – 10 |
| Final assessments  **Risk of MRPs:**  doMESTIC RISK tool  Hospitalisation/ED admissions | 0 – 1 – 2 – 3 – 4 – 5 – 6 – 7 – 8 – 9 – 10  0 – 1 – 2 – 3 – 4 – 5 – 6 – 7 – 8 – 9 – 10 |
| Intervention t1 | 0 – 1 – 2 – 3 – 4 – 5 – 6 – 7 – 8 – 9 – 10 |
| Intervention t2 | 0 – 1 – 2 – 3 – 4 – 5 – 6 – 7 – 8 – 9 – 10 |
| Intervention t3 | 0 – 1 – 2 – 3 – 4 – 5 – 6 – 7 – 8 – 9 – 10 |

**The dose of nursing in the intervention programme (0 = not enough; 1 = no changes necessary; 2 = need to be increased)**

|  | **Amount** | **Frequency** | **Intensity** | **Duration** |
| --- | --- | --- | --- | --- |
| Intervention t1 |  |  |  |  |
| Intervention t2 |  |  |  |  |
| Intervention t3 |  |  |  |  |
|  |  |  |  |  |

**Clinical relevance of the MRP intervention (0 = not all relevant; 10 = completely relevant) (stakeholders’ opinions)**

|  | Older adult | Nurse | Pharmacist | Physician |
| --- | --- | --- | --- | --- |
| Avoiding MRPs | 0 – 1 – 2 – 3 – 4- 5- 6 – 7 – 8 – 9 – 10 | 0 – 1 – 2 – 3 – 4- 5- 6 – 7 – 8 – 9 – 10 | 0 – 1 – 2 – 3 – 4- 5- 6 – 7 – 8 – 9 – 10 | 0 – 1 – 2 – 3 – 4- 5- 6 – 7 – 8 – 9 – 10 |
| Preventing hospitalisation | 0 – 1 – 2 – 3 – 4- 5- 6 – 7 – 8 – 9 – 10 | 0 – 1 – 2 – 3 – 4- 5- 6 – 7 – 8 – 9 – 10 | 0 – 1 – 2 – 3 – 4- 5- 6 – 7 – 8 – 9 – 10 | 0 – 1 – 2 – 3 – 4- 5- 6 – 7 – 8 – 9 – 10 |
| Frailty | 0 – 1 – 2 – 3 – 4- 5- 6 – 7 – 8 – 9 – 10 | 0 – 1 – 2 – 3 – 4- 5- 6 – 7 – 8 – 9 – 10 | 0 – 1 – 2 – 3 – 4- 5- 6 – 7 – 8 – 9 – 10 | 0 – 1 – 2 – 3 – 4- 5- 6 – 7 – 8 – 9 – 10 |
| Remaining at home | 0 – 1 – 2 – 3 – 4- 5- 6 – 7 – 8 – 9 – 10 | 0 – 1 – 2 – 3 – 4- 5- 6 – 7 – 8 – 9 – 10 | 0 – 1 – 2 – 3 – 4- 5- 6 – 7 – 8 – 9 – 10 | 0 – 1 – 2 – 3 – 4- 5- 6 – 7 – 8 – 9 – 10 |
| Interprofessional collaboration | 0 – 1 – 2 – 3 – 4- 5- 6 – 7 – 8 – 9 – 10 | 0 – 1 – 2 – 3 – 4- 5- 6 – 7 – 8 – 9 – 10 | 0 – 1 – 2 – 3 – 4- 5- 6 – 7 – 8 – 9 – 10 | 0 – 1 – 2 – 3 – 4- 5- 6 – 7 – 8 – 9 – 10 |
