# Supplementary material for: An Intervention Program to Reduce Medication-Related Problems Among Polymedicated Home-Dwelling Older Adults (OptiMed): Protocol for a Pre-Post, Multisite, Pilot, and Feasibility Study
Source: JMIR Res Protoc. 2023 Jan 25;12:e39130. doi: 10.2196/39130 (PMC9909524; doi:10.2196/39130)
Supplement: Multimedia Appendix 9 [file resprot_v12i1e39130_app9.docx]

Multimedia Appendix 9. Overview of the OptiMed pre-post and multi-site pilot and feasibility study: an intervention program to optimize medication management and prevent MRPs among polymedicated, home-dwelling older adults with multiple chronic conditions.

| **Participant recruitment** | **Contact with study and collaborating participants (t - 1)** | **Baseline assessment after written consent (preintervention)** | **Medication-management intervention program to reduce the risks of MRPs** | | | **Final assessment (post-intervention)** |
| --- | --- | --- | --- | --- | --- | --- |
|  |  | **Week 0 = measurement 1 (t0)** | **Week 1 (t1)** | **Week 2 (t2)** | **Weeks 3 and 4 (t3)** | **Week 5 = measurement 2 (t4)**  **measurement 1 + 35–40 days** |
| **Study participants:**  Polymedicated home-dwelling older adults at risk of MRPs  Sample pilot study (n = 30)  **Inclusion criteria:**  - Aged 65 years or older, both for men and women  - People with multiple chronic conditions (2 or more)  - Managing at least 4 prescribed medications daily  - At risk of MRPs (score ≥ 5)  - Living alone or with a partner, in a rural or urban area  - Supported by a primary healthcare centre  - An informal caregiver is involved in medication management  **Collaborating participants:**  Informal caregivers  - Designated by the older adult as the most significant informal caregiver involved in their medication management  - Aged 18 years or above  Nurse primary-care manager  - Working in a primary healthcare centre  - Already knows the participant  Community pharmacist  -Study partner or works in a community pharmacy  Physician  - Older adult’s GP  - Designated by the older adult as having a key role in their medication management | Nurse primary-care manager working for a primary healthcare centre, already knows the participant professionally and explains the study to them  In collaboration with the primary healthcare centre:  1. Recruitment of participants  2. First meeting, within 3 to 4 days, at the participant’s home  3. Oral and written information and explanation about the study  4. Written consent | **Older participants** (Multimedia Appendix 5):  - Sociodemographic data  - Health status:  - 6-CIT  - TFI  - ICD-10 diagnosis  - Risk of MRPs:  - Medication list(s)  - Previous MRPs  - doMESTIC risk tool^a^  ** Assessment carried out by two research nurses collaborating with two community pharmacist study partners.*  **Informal caregiver** (Multimedia Appendix 6)**:**   - Sociodemographic data - Role in medication management   **Pharmacist** (Multimedia Appendix 7)**:**   - Sociodemographic and professional data   **Nurse primary-care manager** (Multimedia Appendix 7)**:**   - Sociodemographic and professional data   **Physician** (Multimedia Appendix 7)**:**   - Sociodemographic and professional data   *All data will be accessed using secuTrial^®^.* | Reviewing prescribed medications using the STOPP/START criteria (16):  - By the pharmacist study partner in close collaboration with the older adult’s physician- For older participants at risk of MRPs detected using the doMESTIC risk tool (t0) | Exploring older participant and informal caregiver’s needs and care goals to reduce the older adult’s risks of MRPs:  - One joint consultation between the older participant, their informal caregiver and the research nurse.  Designing a target education plan to empower the older participant and informal caregiver’s medication management and to promote their active engagement in reducing the risks of MRPs (items 9 and 10 from doMESTIC risk tool):  - By the research nurse  - In collaboration (if available) with the designated healthcare professional(s) | Implementing the target education plan to empower older participant and informal caregiver’s medication management and promote their active engagement to reduce risks of MRPs  - Two joint consultations (once a week) between the older participant, the informal caregiver and the research nurse | **Older participant at risk of MRPs (**Multimedia Appendix 1)**:**  - Risk of MRPs with doMESTIC risk tool^a^  - Number and type of medication changes made (by the physician)  - Acceptability assessment (Multimedia Appendix 5)  **Collaborating participants:**  Acceptability assessment (Multimedia Appendixs 6-7)  **Pilot study:**  Feasibility outcomes (Table 2) assessment (Multimedia Appendix 8) |

^a^ Assessment carried out by 2 research nurses collaborating with 2 community pharmacists partnering with the study.

GP: general practitioner

MRP: medication-related problem

TFI: Tilburg Frailty Indicator
