# Supplementary material for: An Intervention Program to Reduce Medication-Related Problems Among Polymedicated Home-Dwelling Older Adults (OptiMed): Protocol for a Pre-Post, Multisite, Pilot, and Feasibility Study
Source: JMIR Res Protoc. 2023 Jan 25;12:e39130. doi: 10.2196/39130 (PMC9909524; doi:10.2196/39130)
Supplement: Multimedia Appendix 10 [file resprot_v12i1e39130_app10.docx]

Multimedia Appendix 10. Timetable for the OptiMed pilot study.

| ***Study tasks*** | ***Month 0*** | ***Month 1*** | ***Month 2*** | ***Month 3*** | ***Month 4*** | ***Month 5*** | ***Month 6*** | ***Month 7*** | ***Month 8*** | ***Month 9*** |
| --- | --- | --- | --- | --- | --- | --- | --- | --- | --- | --- |
| Preparing eCRF (case report form) with secuTrial® |  |  |  |  |  |  |  |  |  |  |
| Meetings with field partners |  |  |  |  |  |  |  |  |  |  |
| Participant recruitment |  | First patient in |  |  | Last patient in |  |  |  |  |  |
| Participant follow-up |  |  |  |  |  | Last patient out |  |  |  |  |
| Intermediate analysis |  |  |  |  |  |  |  |  |  |  |
| Final analysis and writing final paper |  |  |  |  |  |  |  |  |  |  |
